# Complementary anti-cancer pathways triggered by inhibition of sideroflexin 4 in ovarian cancer

**DOI:** 10.1038/s41598-022-24391-3

**Published:** 2022-11-19

**Authors:** Lia Tesfay, Bibbin T. Paul, Poornima Hegde, Molly Brewer, Samrin Habbani, Evan Jellison, Timothy Moore, Hao Wu, Suzy V. Torti, Frank M. Torti

**Affiliations:** 1 Department of Molecular Biology and Biophysics, University of Connecticut Health Center, Farmington, CT 06030 USA; 2 Department of Pathology, University of Connecticut Health Center, Farmington, CT 06030 USA; 3 Department of Obstetrics and Gynecology, University of Connecticut Health Center, Farmington, CT 06030 USA; 4 Department of Immunology, University of Connecticut Health Center, Farmington, CT 06030 USA; 5 Statistical Consulting Services, Center for Open Research Resources, University of Connecticut, Storrs, CT 06269 USA; 6 Department of Statistics, University of Connecticut, Storrs, CT 06269 USA; 7 Department of Medicine, University of Connecticut Health Center, Farmington, CT 06030 USA; 8 Present Address: Department of Comparative Pathobiology, Purdue University College of Veterinary Medicine, West Lafayette, IN 47907 USA

**Keywords:** Cancer, Cell biology, Molecular medicine, Oncology

## Abstract

DNA damaging agents are a mainstay of standard chemotherapy for ovarian cancer. Unfortunately, resistance to such DNA damaging agents frequently develops, often due to increased activity of DNA repair pathways. Sideroflexin 4 (SFXN4) is a little-studied inner mitochondrial membrane protein. Here we demonstrate that SFXN4 plays a role in synthesis of iron sulfur clusters (Fe-S) in ovarian cancer cells and ovarian cancer tumor-initiating cells, and that knockdown of SFXN4 inhibits Fe-S biogenesis in ovarian cancer cells. We demonstrate that this has two important consequences that may be useful in anti-cancer therapy. First, inhibition of Fe-S biogenesis triggers the accumulation of excess iron, leading to oxidative stress. Second, because enzymes critical to multiple DNA repair pathways require Fe-S clusters for their function, DNA repair enzymes and DNA repair itself are inhibited by reduction of SFXN4. Through this dual mechanism, SFXN4 inhibition heightens ovarian cancer cell sensitivity to DNA-damaging drugs and DNA repair inhibitors used in ovarian cancer therapy, such as cisplatin and PARP inhibitors. Sensitization is achieved even in drug resistant ovarian cancer cells. Further, knockout of SFXN4 decreases DNA repair and profoundly inhibits tumor growth in a mouse model of ovarian cancer metastasis. Collectively, these results suggest that SFXN4 may represent a new target in ovarian cancer therapy.

## Introduction

Cancer cells, including breast, prostate, ovarian cancer cells and others, acquire more iron than their normal counterparts, presumably harnessing this iron to sustain metabolic processes that foster tumor growth and metastasis^1^. Increased iron is reflected in an increase in the small but metabolically active iron compartment termed the labile iron pool^1,2^. Through its participation in the Fenton reaction and other pathways, excess iron contributes to the oxidative fragility of cancer cells and heightens cancer cell susceptibility to agents that trigger oxygen radical-mediated cell death^3^. The mechanism of this lethal oxidant injury involves damage to cellular constituents, particularly DNA, by hydroxyl and other radicals^4–6^. Sensitivity to oxidative stress has long been considered an Achilles heel of cancer cells and is now being exploited in clinical trials^7,8^.

Cancer cells exhibit increased endogenous DNA damage when compared to their normal counterparts. This is due not only to elevated oxidative stress, but also to increased proliferation, mutations in tumor suppressor proteins, and multiple other mechanisms^9^. In response to increased endogenous DNA damage, cancer cells upregulate DNA repair pathways, including homologous recombination repair (HRR) and nucleotide excision repair (NER)^9,10^. Recent evidence suggests that many enzymes in DNA processing and repair require iron for their function, frequently in the form of iron-sulfur (Fe-S) clusters^11^. Synthesis of iron-sulfur clusters involves multiple steps, the earliest of which are carried out in mitochondria^12^.

In this manuscript we demonstrate that the little-studied mitochondrial inner membrane protein sideroflexin 4 (SFXN4) is essential to the production of Fe-S cluster proteins in ovarian cancer cells. Targets of SFXN4 disruption include many DNA repair proteins that require Fe-S clusters for their function. In addition, disruption of SFXN4 results in iron accumulation in the mitochondria, leading to enhanced oxidative damage to DNA. Thus, inhibition of SFXN4 simultaneously targets two complementary pathways in cancer cells. First, it augments oxidant stress through iron accumulation, leading to DNA damage. Second, it disrupts Fe-S clusters in DNA repair pathways, leading to reduced DNA repair capacity. These metabolic alterations reduce proliferation of ovarian cancer cells and sensitizes them to both cisplatin and poly-ADP ribose polymerase (PARP) inhibitors. Further, disruption of SFXN4 leads to a marked reduction in cancer growth and metastases in a murine model of peritoneal ovarian cancer. SFXN4 may represent a new target in anti-cancer therapy.

## Results

### Sideroflexin 4 is overexpressed in ovarian cancer tissue and ovarian cancer cell lines

**Figure 1:**
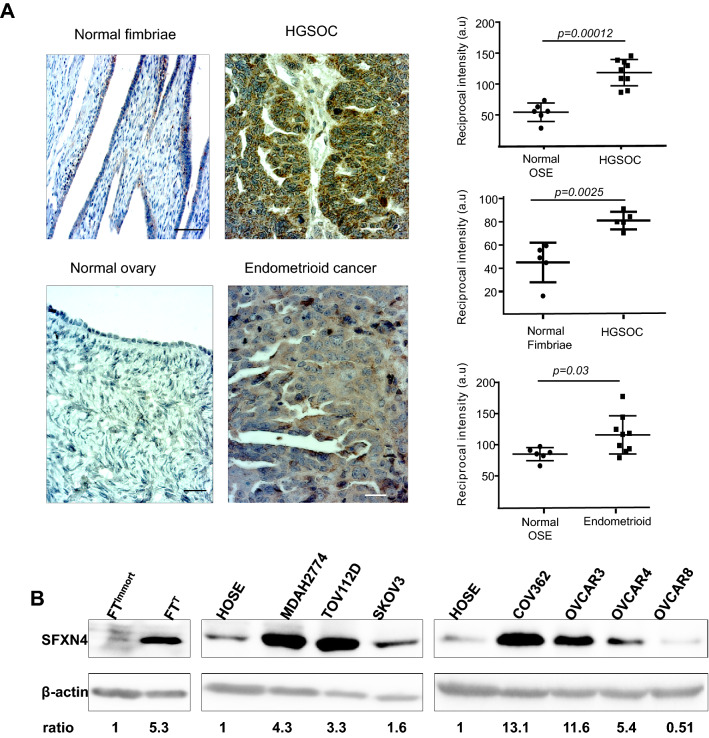
SFXN4 is over expressed in ovarian cancer and ovarian cancer cell lines. (**A**) Representative images of immunohistochemical staining of normal fimbriae and ovarian surface epithelium (OSE), HGSOC, and endometrioid adenocarcinoma stained with anti-SFXN4 antibody. Dot plots represent quantification of staining of tissues collected from 9 patients with HGSOC and endometrioid adenocarcinoma compared to 6 subjects with normal ovarian surface epithelium and five with normal fimbriae. Images of three to four random fields per slide were quantified; each dot represents the average value obtained for an individual patient. Scale bar is 50 µm. (**B**) Immunoblot of SFXN4 from immortalized fallopian tube cells (FT^immort^), transformed fallopian tube cells (FT^T^), human ovarian surface epithelial (HOSE) cells and the indicated ovarian cancer cells representing different subtypes: endometrioid adenocarcinoma (MDAH2774 and TOV112D), adenocarcinoma (SKOV3) and high-grade serous carcinoma (COV362, OVCAR3, OVCAR4 and OVCAR8). Uncropped images of western blots are presented in Supplemental data (Supplemental Fig. S9).

To explore levels of expression of SFXN4 in human ovarian cancer, we first performed immunohistochemical staining of SFXN4 in tissue obtained from ovarian cancer patients. Ovarian cancer comprises multiple histological subtypes. The most prevalent are surface epithelial cancers, including high grade serous ovarian cancer (HGSOC), the most common and lethal subtype, as well as endometrioid and other less common histological subtypes^13^. Immunohistochemical staining with an anti-SFXN4 antibody demonstrated a striking and statistically significant increase in SFXN4 staining in both HGSOC and endometrioid tumor tissue when compared to normal ovarian surface epithelium and fimbriae of the fallopian tube, tissues of origin of these cancers^14^ (Fig. 1A).

We next utilized a series of ovarian cancer cell lines to explore consequences of modulating levels of SFXN4. We used western blotting to assess levels of SFXN4 in multiple ovarian cancer cell lines and normal human ovarian surface epithelial cells (HOSE). With the exception of OVCAR8, SFXN4 was up-regulated in all ovarian cancer cell lines examined, including cell lines representative of HGSOC (COV362, OVCAR3 and OVCAR4); endometrioid (MDAH2774 and TOV112D) and ovarian adenocarcinoma (SKOV3) (Fig. 1B). Notably, this included FT^T^ cells, a genetic model of ovarian cancer tumor-initiating (“stem”) cells created by transduction of normal fallopian cells with SV40 large T, hTERT and c-myc^15^, which we compared to its immortalized counterpart (FT^immort^) transduced with SV40 large T and hTERT alone.

### SFXN4 knockdown impairs Fe-S biogenesis, inhibits mitochondrial respiration, and increases mitochondrial ROS in ovarian cancer cells

**Figure 2:**
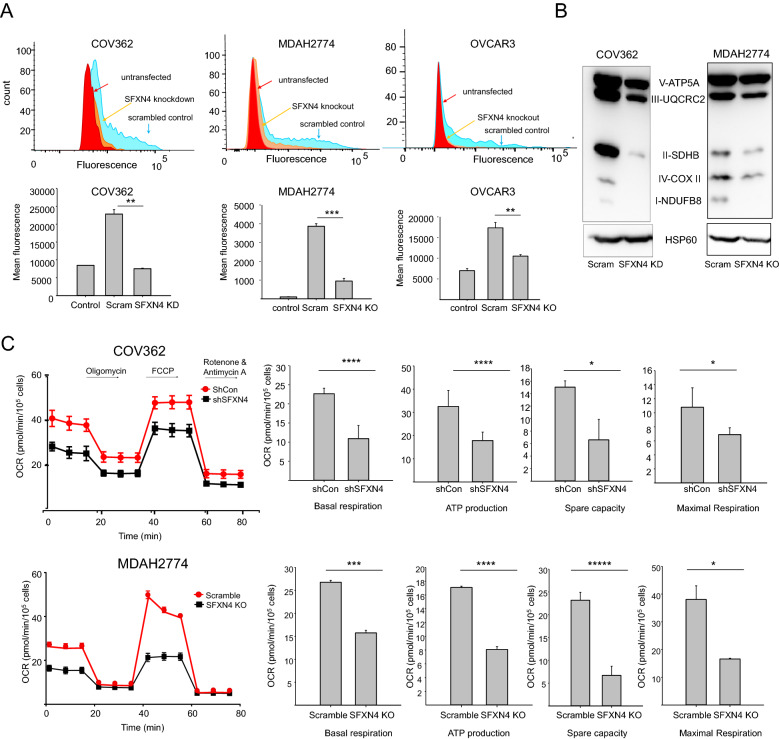
Depletion of SFXN4 reduces Fe-S cluster assembly, mitochondrial respiratory complex proteins, and mitochondrial respiration. (**A**) Relative fluorescence of GRX2-Fe-S dependent sensors in untransfected, scrambled controls, and SFXN4 knockdown COV362, MDAH2774 and OVCAR3 cells. Panels below are quantification of mean fluorescence intensity in three independent Fe-S cluster formation experiments with **p < 5.6E−04; ***p < 1.3E−05. (**B**) Western blots showing levels of labile subunits from each of the five mitochondrial respiratory complexes in COV362 and MDAH2774 SFXN4 KO and control cells. Uncropped images are presented in Supplemental data (Supplemental Fig. S9). (**C**) Changes in oxygen consumption rate in response to treatment with indicated metabolic inhibitors in COV362 (above) and MDAH2774 (below) SFXN4 KD or SFXN4 KO and control cells. Panels at right show basal respiration, mitochondrial ATP production, maximal respiration, and spare capacity quantified in 3–12 replicate experiments (means and standard deviation with *p < 1.2E−02; ***p < 1.3E−05; ****p < 3.8E−06; ******p < 1.8E−13).

**Figure 3:**
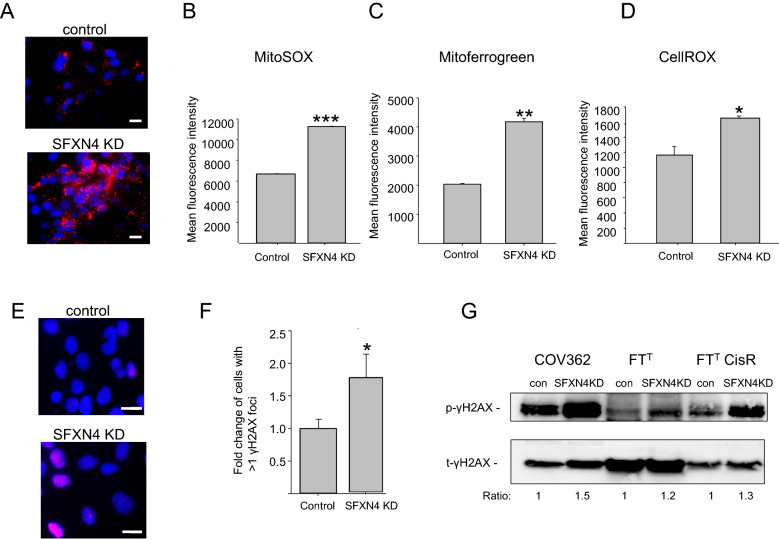
SFXN4 KD increases mitochondrial superoxide, mitochondrial iron, cellular ROS and p-γH2AX. (**A**) COV362 control and SFXN4 knockdown cells were stained with MitoSOX and visualized using fluorescence microscopy (**B**) quantification of MitoSOX positive cells in control and SFXN4 knockdown cells. (**C**) Quantification of mitochondrial iron measured by mitoferrogreen staining in control and siSFXN4 knockdown cells. (**D**) Cellular reactive oxygen species in control and SFXN4 knockdown cells quantified using CellROX. (**E**) Representative image of COV362 control and SFXN4 knockdown cells stained with p-γH2AX antibody (red) and counterstained with Dapi (blue). (**F**) Quantification of γH2AX foci. (**G**) Western blot analysis of p-γH2AX and t-γH2AX in control and SFXN4 knockdown cells. Uncropped images are presented in Supplemental data (Supplemental Fig. S10). Scalebar in (**A**) and (**E**) is 10 µm. Means and standard deviations of three independent experiments (****p < 1.5E -09; ***p < 2.5E−05; **p < 0.002; *p < 0.026).

We previously showed that knockdown of SFXN4 reduces Fe-S cluster biogenesis and mitochondrial respiration in hematopoetic cells^16^. To test whether these effects were also seen in ovarian cancer cells, we first directly measured Fe-S cluster formation using a validated Fe-S cluster formation assay in which cells are transfected with two separate vectors, one encoding the N-terminal half of Venus fluorescent protein fused to the Fe-S-containing protein glutaredoxin2 (GRX2), and the second encoding the C-terminal half of Venus fluorescent protein fused to GRX2^17^. The fluorescence observed when these two fusion proteins are co-expressed is contingent on GRX2 homodimerization, a process that depends quantitatively on the availability of [2Fe-2S] clusters. Using this assay, we first showed that Fe-S formation was significantly reduced following knockdown of SFXN4 in both HGSOC cells (COV362 and OVCAR3) and endometrioid (MDAH2774) ovarian cancer cells (Fig. 2A). Second, we used western blotting to assess levels of mitochondrial respiratory enzymes that contain Fe-S clusters, since these proteins are typically degraded if Fe-S clusters are not inserted into the cognate apoproteins^18^. As seen in Fig. 2B, levels of mitochondrial respiratory proteins were also reduced following SFXN4 knockdown. Third, we used the Agilent Seahorse XF Cell Mito Stress Test assay to measure mitochondrial respiration, which was significantly reduced by knockdown of SFXN4, consistent with the reduction in respiratory proteins that contain Fe-S clusters (Fig. 2C). Fourth, we examined levels of mitochondrial superoxide, since this byproduct of mitochondrial oxidative phosphorylation becomes elevated when respiration is impaired^19^. Knockdown of SFXN4 resulted in a significant elevation of mitochondrial superoxide in both COV362 HGSOC (Fig. 3A,B) and endometrioid MDAH2774 (Supplemental Fig. S1) ovarian cancer cell lines. Thus, SFXN4 knockdown impairs Fe- S cluster formation and increases mitochondrial reactive oxygen species in ovarian cancer cells.

### Targeting SFXN4 increases mitochondrial iron and oxidative stress and triggers a DNA damage response

Impaired Fe-S cluster formation typically results in mitochondrial iron overload, although the mechanism underlying this response is incompletely understood^20^. To assess the impact of SFXN4 on mitochondrial iron, we used Mito-FerroGreen™, a fluorescent probe that detects reduced iron (Fe^+^^2^) in mitochondria. Consistent with our observation of impaired Fe-S cluster formation following knockdown of SFXN4, we observed a significant increase in mitochondrial iron in HGSOC cells in which SFXN4 had been knocked down (Fig. 3C). Since Fe^2+^ is a well-known Fenton catalyst, this result suggested that SFXN4 knockdown might also increase overall cellular oxidative stress. To test this hypothesis, we assessed cellular reactive oxygen species using CellROX™, a cell-permeant fluorogenic probe that fluoresces upon oxidation by reactive oxygen species. As shown in Fig. 3D and Supplemental Fig. S1, this probe revealed a significant increase in cellular ROS following knockdown of SFXN4.

Oxygen radicals produced in cells undergoing oxidative stress can damage DNA through production of abasic sites as well as oxidative modification of DNA bases and DNA strand breaks, triggering a DNA damage response that includes the activation and recruitment of phosphorylated histone γH2AX to sites of DNA damage^21^. We therefore tested whether knockdown of SFXN4 resulted in DNA damage by staining for γH2AX. As shown in Fig. 3E,F, there was a statistically significant increase in nuclear staining for γH2AX in SFXN4 knockdown cells compared to controls. We confirmed this result by western blotting for phosphorylated γH2AX, which similarly showed an increase in the ratio of phosphorylated to total γH2AX following knockdown of SFXN4 in COV362 HGSOC cells as well as in FT^T^ovarian cancer stem cells and FT^T^ CisR cisplatin-resistant ovarian cancer stem cells (Fig. 3G). Collectively, these results indicate that impaired formation of Fe-S clusters and enhanced oxidative stress following SFXN4 knockdown are sufficient to induce a DNA damage response.

### Knockdown of SFXN4 decreases DNA repair

We next explored whether SFXN4 knockdown might also compromise DNA repair. Among the proteins dependent on Fe-S clusters for their function are enzymes critical to DNA replication and repair of damaged DNA. These include DNA polymerases such as DNA polymerase delta (*POLD1*)^22^, helicases such as XPD (*ERCC2*)^23^ and FANCJ (*BRIP1/BAC*)^24,25^, and glycosylases such as endonuclease III-like protein 1 (*hNTH1)*^26^ and adenine DNA glycosylase (*MUTYH*)^27^. These proteins function in multiple DNA repair pathways including base excision repair, nucleotide excision repair, and double strand break repair. Mutations in several of these proteins have been directly linked to drug sensitivity: for example, XPD and polymerase delta are both involved in nucleotide excision repair (NER)^22^, a pathway that has been linked to sensitivity to cisplatin in a number of malignancies^22,28,29^, including ovarian cancer^30,31^. Similarly, mutations in FANCJ, a protein that physically interacts with breast cancer type 1 susceptibility protein (*BRCA1*)^32^ and plays a key role in homologous recombination repair (HRR)^24^, increase sensitivity to PARP inhibitors^33^.

**Figure 4:**
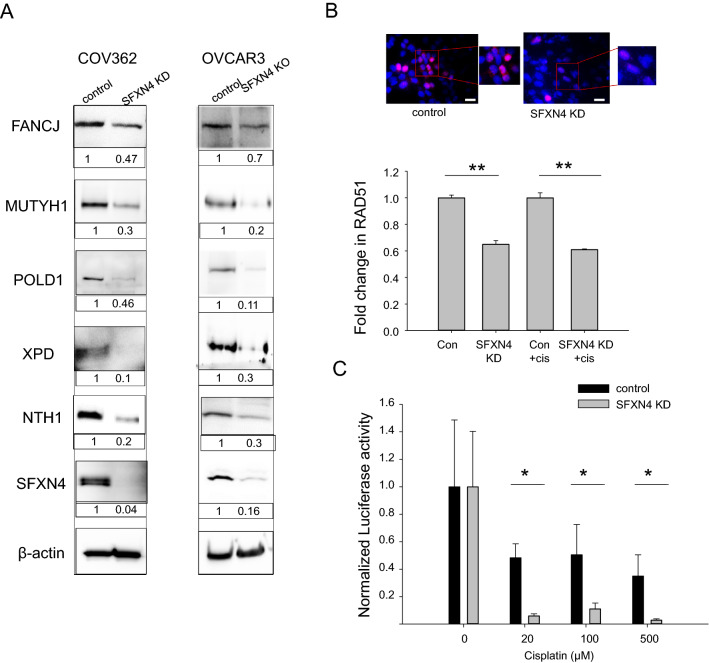
SFXN4 KD decreases Fe-S containing DNA repair proteins, RAD51, and DNA repair capacity. (**A**) Western blot of Fe-S containing DNA repair proteins in control and SFXN4 knock-down ovarian cancer cells (siRNA was used to knock down SFXN4 in COV362 cells and CRISPER/Cas was used to knockout SFXN4 KO in OVCAR3 cells). Uncropped images are presented in Supplemental data (Supplemental Figs. S11, S12). (**B**) Representative immunofluorescence image of RAD51 foci (red) in siRNA SFXN4 knock-down and siRNA control COV362 cells (nuclei were stained with DAPI) and quantification of fold change in RAD51 foci in SFXN4 knockdown versus control cells treated without or with 20 µM cisplatin for 48 h (means and standard deviations, **p < 6.4E−05). Scalebar is 10 µm. (**C**) DNA repair capacity as measured by dual luciferase assay in siRNA control and siSFXN4 COV362 cells transfected with undamaged or cisplatin damaged luciferase plasmid for 24 h. Shown are means and standard deviations of 3 independent experiments, each with 4 replicas (*p < 4E−04).

As shown in Fig. 4A and Supplemental Fig. S2, SFXN4 knockdown decreased levels of all these Fe-S-containing proteins in HGSOC ovarian cancer cell lines (COV362, OVCAR3), ovarian cancer stem cells (FT^T^), and endometrioid cancer cells (MDAH2774), with the reduction ranging from 30 to 90%, as assessed by western blotting. Reduction in levels of SFXN4 did not affect non-Fe-S containing proteins, including GAPDH, tubulin, the mitochondrial protein AFG3L2 and DNA binding proteins C/EBPβ, ID2 and RPA32 (Supplemental Fig. S2). Rescue experiments demonstrated that levels of Fe-S proteins were restored following re-introduction of SFXN4, ruling out off-target effects (Supplemental Fig. S3).

To test whether these decreases in DNA repair proteins resulted in the predicted reduction in DNA repair capacity (i.e., to eliminate the possibility that reduction in one pathway was compensated for by an increase in another pathway), we first assessed nuclear recruitment of RAD51, a DNA recombinase involved in repair of DNA double strand breaks^34^, to evaluate the effect of SFXN4 knockdown on the repair response to DNA damage^35,36^.

Depletion of SFXN4 significantly reduced RAD51 staining in nuclei under basal conditions, consistent with reduced basal levels of DNA repair following SFXN4 knockdown (Fig. 4B). We then treated cells with cisplatin to induce DNA damage and compared the recruitment of RAD51 to nuclei in control and SFXN4 knockdown cells treated with cisplatin. RAD51 staining remained significantly lower in SFXN4 knockdown cells than in control cells (Fig. 4B). Thus, SFXN4 knockdown impairs repair as assessed by recruitment of RAD51 to damaged DNA.

To confirm this result, we directly measured the effect of SFXN4 on DNA repair capacity using a host cell reactivation assay^37^. In this assay, a plasmid encoding Firefly luciferase was treated ex vivo with cisplatin or vehicle to induce DNA damage, followed by transfection into control cells or SFXN4 knockdown cells and measurement of luciferase activity. Transfection with an undamaged Renilla luciferase vector was used as an internal control for transfection efficiency. As seen in Fig. 4C, knockdown of SFXN4 significantly reduced DNA repair capacity as measured by this functional assay. Thus, knockdown of SFXN4 decreases basal levels of DNA repair as well as repair of DNA damage induced by the chemotherapeutic drug cisplatin.

### SFXN4 inhibition enhances the response of ovarian cancer and ovarian cancer stem cells to cisplatin and PARP inhibitors

Drugs used in the treatment of ovarian cancer include cisplatin and carboplatin, drugs that have been the mainstays of ovarian cancer therapy for decades, as well as the more recently developed PARP inhibitors^13^. Our observation that SFXN4 knockdown decreased DNA repair suggested that SFXN4 knockdown might sensitize ovarian cancer cells to these clinically relevant chemotherapeutic drugs.

**Figure 5:**
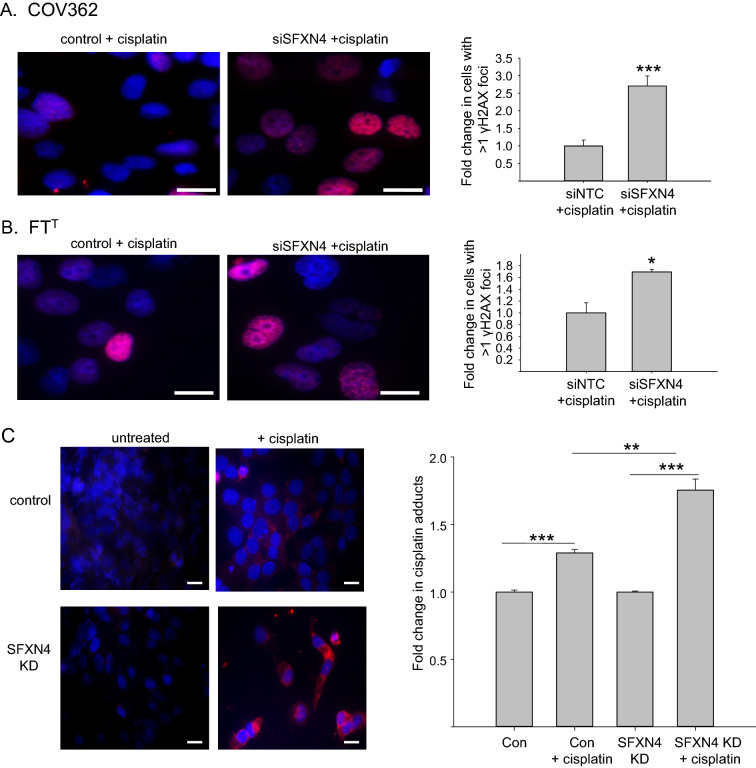
Knockdown of SFXN4 increases γ-H2AX focus formation and the accumulation of cisplatin adducts in ovarian cancer cells. (**A**) Left: Representative immunofluorescent image of COV362 cells treated with non-targeting or SFXN4 siRNA and cisplatin and stained for γH2AX; right: quantification of 3 independent experiments. (**B**) γH2AX staining of FT^T^ control and SFXN4 knockdown cells. **(C**) Left: immunofluorescence images of cisplatin adducts in COV362 cells treated with non-targeting or SFXN4 siRNA. Cells were left untreated or exposed to cisplatin prior to staining with anti-human cisplatin modified DNA antibody (red) and counterstaining with Dapi (blue). Right: cisplatin adducts were quantified using flow cytometry. Shown are means of three replicas from three independent experiments. Scale bars are 10 µm. *p < 2.5E−03; **p < 7E−04; ***p < 9E−05.

To test this possibility, we first measured the effect of SFXN4 on DNA damage in cisplatin-treated cells using γH2AX staining, the same assay we had used to evaluate DNA damage following SFXN4 knockdown. As shown in Fig. 5A,B, in both COV362 HGSOC cells and in FT^T^ ovarian cancer stem cells, SFXN4 knockdown significantly increased DNA damage following cisplatin treatment, consistent with the ability of SFXN4 knockdown to attenuate DNA repair. We corroborated these results by assessing the abundance of cisplatin DNA adducts in cells treated with cisplatin, reasoning that impaired DNA repair would enable these adducts to accumulate more readily. As shown in Fig. 5C, these predictions were confirmed: staining with fluorescent antibody to cisplatin adducts was observed following treatment of cells with cisplatin, whereas untreated cells exhibited no detectable fluorescence (Fig. 5C). Quantification of these results demonstrated that SFXN4 knockdown significantly increased the number of adducts following treatment with cisplatin when compared to cisplatin-treated control cells (Fig. 5C).

We next tested whether the observed increase in cisplatin adducts in SFXN4 knockdown cells would be reflected in enhanced sensitivity to cisplatin. Control and SFXN4 knockdown cells were treated with various concentrations of cisplatin, viability assessed using calcein staining, and an IC50 calculated using the CompParm function in R (see Materials and Methods). As shown in Table 1 and Supplemental Fig. S4, SFXN4 knockdown resulted in a consistent reduction in the cisplatin IC50 (i.e. enhanced cisplatin sensitivity). Since PARP inhibitors represent another promising chemotherapeutic in the treatment of patients with ovarian cancer, we also assessed whether SFXN4 knockdown would sensitize ovarian cancer cells to the PARP inhibitor niraparib. Sensitivity to this drug was similarly enhanced in SFXN4 knockdown cells (Table 1).

**Table 1:** Effect of SFXN4 knockdown on drug sensitivity of ovarian cancer cells.

Cell type	Drug	Ratio of IC50 values in SFXN4 knockdown or SFXN4 knockout* cells versus control (SE)			Average ratio	Average percent reduction in IC50 by SFXN4 KD**
		Expt 1	Expt 2	Expt 3		
COV362	Cisplatin	0.52 (0.06)	0.40 (0.13)	0.41 (0.07)	0.47	53
	Rucaparib	0.64 (0.11)	0.76 (0.10)	0.76 (0.06)	0.74	26
A2780CisR	Cisplatin	0.69 (0.07)	0.44 (0.04)	0.27 (0.18)	0.49	51
	Rucaparib	0.81 (0.04)	0.69 (0.04)	0.83 (0.06)	0.77	23
MDAH2774	Cisplatin	0.23 (0.05)	0.70 (0.09)	3.32 (2.64)	0.34	66
	Rucaparib	0.87 (0.05)	0.66 (0.09)	0.65 (0.08)	0.79	21
FT^T^	Cisplatin	0.65 (0.12)	0.95 (0.16)	0.65 (0.09)	0.70	30
	Rucaparib	0.58 (0.05)	0.28 (0.08)	0.31 (0.05)	0.42	58
FT^T^CisR	Cisplatin	0.60 (0.04)	0.70 (0.07)	0.22 (0.02)	0.36	64
	Rucaparib	0.68 (0.07)	0.47 (0.09)	0.49 (0.04)	0.54	46

*SFXN4 levels were reduced by knockout in MDAH2774 and FT^T^ cells and by siRNA knockdown in other cells.

**Percent reduction in IC50 by SFXN4 knockdown or knockout was statistically significant (p < 0.0001) in all cases.

Cancer stem cells and drug resistant cells are believed to be two important contributors to the development of treatment-refractory ovarian cancer^2^. We therefore tested whether SFXN4 knockdown would sensitize cancer stem cells and drug resistant ovarian cancer cells to drug treatment. To test this hypothesis we used FT^T^ cells as a model of stem-cell derived ovarian cancer^15^ as well as A2780Cis cells, an established model of cisplatin resistant ovarian adenocarcinoma cells^38^. We also created a drug resistant model of FT^T^cells (termed FT^T^ CisR) using long-term culture of cells in low concentrations of cisplatin, as previously described for other cell types^38^. The IC50 of cisplatin in both A2780CisR and FT^T^cisR was increased two- to threefold compared to their respective parental cells (IC50 A2780, 26 ± 10 μM; A2780CisR, 69 ± 24 μM; IC50 FT^T^ 16 ± 4.4, FT^T^CisR 53 ± 13, means and S.E., n = 3), and was due at least in part to increased repair (^38^ and Supplemental Fig. S5). We tested the effect of SFXN4 knockdown on these models of ovarian cancer stem cells and drug resistant ovarian cancer cells. As shown in Table 1, SFXN4 knockdown enhanced the sensitivity of both lines to cisplatin and rucaparib. Thus, disruption of SFXN4 enhances the efficacy of platinum-based compounds and PARP inhibitors in a variety of ovarian cancer cell lines, including some with no known functional mutations in BRCA1/2 (A2780Cis, MDAH2774 (Table 1)).

### Knockdown of SFXN4 inhibits tumor formation and growth in vivo

The ability of SFXN4 knockdown to induce oxidative stress (Fig. 3) and decrease DNA repair (Fig. 4) suggested that knockdown of SFXN4 might inhibit tumor growth. To examine this prediction, we used FT^T^ cells, which model stem-cell derived ovarian cancer and give rise to tumors exhibiting the major hallmarks of HGSOC when injected into the peritoneal cavity, including the formation of widely disseminated and rapidly growing tumors^2,15^. We used CRISPER/Cas to knock out SFXN4 in FT^T^ cells. FT^T^ cells prepared using scrambled gRNA were used as a control. In vitro, we observed an approximately 40% reduction in cell number in SFXN4 knockout cells compared to controls over time (Supplemental Fig. S6). A similar modest but appreciable reduction in viability was observed following knockdown of SFXN4 in COV362 ovarian cancer cells (Supplemental Fig. S7). Reduced cell numbers were largely attributable to a decrease in proliferation rather than the induction of cell death, since the fraction of dead cells in the population remained less than 10% (Supplemental Fig. S7). Intriguingly, knockdown of SFXN4 had little effect on normal human ovarian surface epithelial (HOSE) cells, which express lower basal levels of SFXN4 (Fig. 1 and Supplemental Fig. S8).

**Figure 6:**
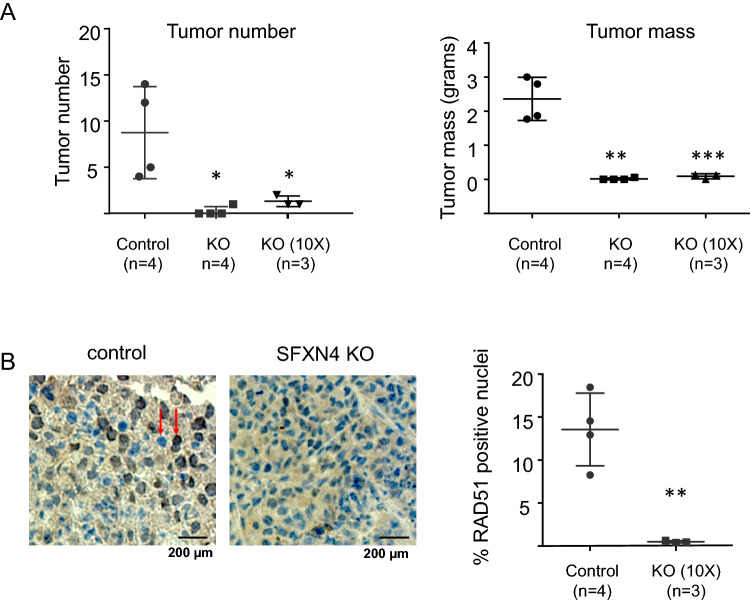
Knockout of SFXN4 reduces tumor number and weight and decreases RAD51 staining in an ovarian cancer metastatic model. (**A**) Control or SFXN4 knockout FT^T^ ovarian cancer stem cells were injected into the mouse peritoneal cavity and tumor number and mass assessed 23 days later. Control cells were injected at 2 × 10^5^ cells/mouse (n = 4) and SFXN4 knockout cells were injected at either the same dose (2 × 10^5^ cells/mouse, n = 4) or tenfold higher (2 × 10^6^ cells/mouse—10 ×; n = 3). (**B**) Tumors were excised from control mice or SFXN4 KO mice and stained for RAD51. Left: representative images with examples of positive and negative nuclear staining indicated (arrows), scale bar is 200 µm; right: average percent RAD51 positive nuclei per tumor *p < 0.05; **p < 0.005; ***p < 0.0005.

To assess the effects of SFXN4 knockout on tumor formation and growth in vivo, we injected both SFXN4 knockout and control FT^T^ cells intraperitoneally in mice and measured tumor size and number approximately 3 weeks later. As shown in Fig. 6A, knockout of SFXN4 led to a dramatic reduction in both tumor number and size, even when the tumor cell inoculum was increased tenfold (Fig. 6A).

Although the remarkable efficacy of SFXN4 knockout in inhibiting tumor growth in vivo precluded assessment of the combined effects of drug treatments and SFXN4 inhibition in vivo*,* immunohistochemical staining of tumors revealed that nuclear RAD51 was lower in SFXN4 knockout tumors than in controls (Fig. 6B). This result is consistent with our in vitro observations (Fig. 4) and indicates that DNA repair is impaired by loss of SFXN4 in vivo as well as in vitro. Thus, targeting SFXN4 inhibits tumor growth and also exhibits the potential to reduce repair and thereby enhance the response to DNA-damaging agents in vivo.

## Discussion

In this manuscript, we demonstrate that SFXN4, a protein that is up-regulated in ovarian cancer (Fig. 1), plays an important role in Fe-S cluster formation in ovarian cancer cells (Fig. 2). Further, we show that the decrease in Fe-S cluster formation that ensues following knockdown or knockout of SFXN4 targets two distinct but interdependent pathways that can directly or indirectly inhibit ovarian cancer cell growth.

First, inhibition of Fe-S cluster formation leads to the accumulation of iron in mitochondria, which in turn increases oxygen free radical generation and DNA damage (Fig. 3). Since Fe-S cluster proteins are critical for respiratory activity, knockdown of SFXN4 also impairs respiratory activity and ATP generation (Fig. 2). Accordingly, knockdown of SFXN4 reduces proliferation (Supplemental Figs. S6, S7) and markedly and significantly reduces tumor number and mass in a mouse model of ovarian cancer peritoneal metastasis (Fig. 6). Intriguingly, the almost complete inhibition of tumor growth observed in vivo (Fig. 6) was more dramatic than the approximately 40% reduction in tumor cell growth seen in vitro (Supplemental Fig. S6), suggesting that SFXN4 knockdown may exert additional effects that are only apparent in an in vivo context, such as the interaction of tumor cells with the microenvironment.

Second, targeting SFXN4 reduces the activity of DNA repair proteins that require Fe-S clusters for their function and stability, including FANCJ, XPD and DNA polymerase delta (Fig. 4). These proteins are critical to the homologous recombination repair (HRR) and nucleotide excision (NER) DNA repair pathways, which are central to the repair of cisplatin and PARP inhibitor-mediated DNA damage^39^. Accordingly, we observed that disruption of SFXN4 decreases DNA repair in vitro (Fig. 4) and in vivo (Fig. 6) and increases the efficacy of platinum-based compounds and PARP inhibitors in a variety of ovarian cancer cell lines, including some with no known functional mutations in BRCA1/2 (A2780Cis, MDAH2774 (Table 1). Since the degree and duration of response to PARP inhibitors are greatest in ovarian cancer patients who have mutations in DNA repair enzymes (BRCA1/2 and others), the ability of SFXN4 inhibition to simultaneously disable multiple DNA repair pathways suggests that targeting SFXN4 has the potential to extend the excellent response to PARP inhibitors to women without defects in DNA repair enzymes.

Why target SFXN4 instead of inhibiting an enzyme in a critical DNA repair pathway? Inhibiting proteins in a single DNA repair pathway frequently results in a shift to compensatory pathways to maintain DNA repair^10^. Thus, overcoming drug resistance that results from augmented DNA repair has remained challenging, and has limited the effectiveness of anti-cancer approaches that target a single DNA repair enzyme^10^. However, because Fe-S cluster proteins such as XPD, polymerase delta, and FANCJ, which are down-regulated in the absence of SFXN4 (Fig. 4), are essential to the function of both NER and HRR repair, targeting SFXN4 may permit the simultaneous inhibition of multiple DNA repair pathways that contribute to resistance to DNA damaging drugs, thereby limiting the ability of ovarian cancer cells to restore DNA repair by shifting to compensatory repair pathways.

Our data and the literature provide indirect evidence that inhibition of SFXN4 may be tolerated by non-cancer cells, at least over the comparatively short timeframes involved in patient treatment. For example, patients with Friedreich’s Ataxia, a condition caused by mutations in frataxin, a protein involved in iron-sulfur cluster biogenesis, typically remain asymptomatic for a decade or more^40^. Mutations (including frameshift mutations) in SFXN4 have been identified in pediatric patients who did not seek advanced medical care until the ages of 6 and 14 years^41^. Consistent with these observations, we observed little effect of SFXN4 knockdown on viability of normal human ovarian surface epithelial cells (Supplemental Fig. S8).

Although the reason(s) underlying the differential response of cancer and non-cancer cells to inhibition of SFXN4 remain to be further explored, several mechanisms may be involved. Cancer cells, including ovarian cancer cells, generally contain elevated levels of labile iron relative to their normal counterparts^1,2^, rendering them more sensitive to oxidative stress, such as that induced by SFXN4 knockdown. In addition, a selective vulnerability to Fe-S inhibition has been observed in basal-like breast cancer, where it was linked to polymerase epsilon dependence mediated by hyperactive CDK2^42^. We also observed that HOSE cells express lower levels of SFXN4 than cancer cells (Fig. 1 and Supplemental Fig. S8), and it is possible that non-cancer cells may rely on alternative proteins to facilitate Fe-S cluster formation.

A woman diagnosed today with advanced ovarian cancer has only a moderately improved chance of long-term survival compared to a woman diagnosed forty years ago. This is in part because the most effective and widely used drugs, the platinum compounds (cisplatin and carboplatin), lose their effectiveness over time—drug resistance develops. An important (but not the only) reason for resistance to cisplatin and related drugs is an acquired ability of ovarian cancers to repair the DNA damage caused by cisplatin compounds^43^. Targeting the mitochondrial protein sideroflexin 4 (SFXN4) may provide a path to addressing this major limitation to effective ovarian cancer therapy. Exploration of SFXN4 as a target of anti-cancer therapy may deserve consideration.

## Materials and methods

### Cell culture

MDAH2774, SKOV3, TOV-112D cells were purchased from ATCC (on March 6th 2013) and cultured in 10% FBS (Gemini Bio-Products) in DMEM (GIBCO). A2780 and A2780Cis were purchased from Sigma (on February 2017) and cultured in RPMI 1640 + l-glutamine (GIBCO) supplemented with 10% FBS (Gemini Bio-Products). Cells were frozen at low passage and used within 2–3 months after thawing. COV362 cells were purchased from Sigma on May 18th 2015 and cultured in DMEM (GIBCO) containing 10% FBS. FT^T^ and FT^i^ cells (described in^15^) were cultured in DMEM containing 10% FBS. Human Ovarian Surface Epithelial (HOSE) cells (ScienCell Research Laboratories) were cultured in Ovarian Epithelial Cell Medium (ScienCell Research Laboratories). OVCAR-3, OVCAR-4 and OVCAR-8 cells were obtained from NCI (distributed by Charles River Labs) on February 25th 2018. These cells were cultured in RPMI 1640 + l-glutamine (GIBCO) supplemented with 10% FBS (Gemini Bio-Products). All cells were maintained at 37 °C in a humidified incubator at 5% CO_2_.

### Generation of cisplatin-resistant FT^T^ (FT^T^CisR)

FT^T^CisR cisplatin-resistant cells, were obtained by exposing FT^T^ cells to increasing cisplatin concentrations in a stepwise manner, essentially as described^38^.

### Gene silencing by siRNA, shRNA and CRISPR

Transient siRNA-mediated knockdown was performed with ON-TARGETplus siRNAs (humanSFXN4: L-018237-02-0005; NFS1: L-011564-01-0005; and Non-Targeting siGENOME siRNA Pool #2) obtained from Dharmacon. Lentiviral shRNA vector was used to knockdown SFXN4 in COV362 and MDAH2774 cells. The control vector (pGFP-C-shLenti (TR30021)) and shSFXN4 vector (pGFP-SFXN4-shLenti (TL301736)) were obtained from OriGene Technologies Inc., Rockville, MD, USA. The target sequence CTGTGATCTTCCTCGTGCAAGCCAGTGGA is in the SFXN4 coding region. Lentiviral particles were made by transient co-transfection of shRNA vector and packaging vectors (VSVG, pMDLG and RSV-REV) into HEK293T cells. Cells were infected and selected for puromycin resistance for two weeks. For clustered regularly interspaced short palindromic repeats (CRISPR) knock out, guideRNAs were designed to specifically cut in the first exon of the human SFXN4 gene (Ensemble sequence ENSG00000183605). Guide RNA sequence for SFXN4 was GTGATCCAGAAGCGCACGTT and for scrambled control was CAGTCGGGCGTCATCATGAT. Oligos were purchased from Integrated DNA Technologies Inc. (IDT), phosphorylated, and annealed. The annealed gRNA oligos were cloned into the lentiCRISPRv2 plasmid. Lentivirus was made using the packaging plasmids psPAX2 and pMD2.G (Addgene plasmid #12260 and Addgene plasmid #12259) into HEK293T cells using Lipofectamine 2000 (Thermo Fisher). After selection for puromycin resistance, cells were tested using a T7E1 assay (NEB) to verify the creation of insertions or deletions (INDELs). Single colonies were isolated and the region of CRISPR binding was amplified by PCR and sequenced by Sanger sequencing^44^. Clones with frameshift mutations in both alleles were expanded, and knockout of SFXN4 was confirmed by western blot.

### Immunohistochemistry

Sections of formalin-fixed paraffin-embedded (FFPE) de-identified human tissues from HGSOC (9 patients), endometroid (9 patients), normal ovary (6 subjects) and normal fimbriae (obtained from 5 of the 9 HGSOC patients for which normal fimbriae were available) were obtained from the biorepository of UCHC (IRB IE-08-310-1). Tissues were immunostained with antibodies to human SFXN4 (Sigma cat# HPA020872) followed by anti-rabbit secondary antibody (Biocare Medical MACH2 Rabbit HRP-Polymer; catalog # RHRP520#) and staining with 3,3'-diaminobenzidine (Biocare Medical). Gill’s Hematoxylin III and Lithium Blue (Poly Scientific) were used as counterstains. Images of three to four random fields per slide (each field containing approximately 150–200 cells) were quantified as described^45^; each dot in the figure represents the average value obtained for an individual subject. Data is presented as relative intensity units; thus, only samples analyzed in the same staining session are compared (i.e., normal fimbriae vs HGSOC; normal OSE vs HGSOC; and normal OSE vs endometrioid were each stained and quantified separately). A board-certified pathologist (P.H.) provided histomorphologic confirmation of the ovarian cancer subtypes and normal adnexal tissues and their corresponding immunostains that were quantified in this analysis.

### Western blotting

Western blotting was performed as previously described^46^. Electron transport proteins were detected using a total OXPHOS human western blot antibody cocktail (ab110411) from Abcam. The primary antibodies used are SFXN4 (Thermofischer (PA5-35980)), P-Histone H2A.X (Cell Signaling Technology (9718S)), Histone H2A.X (Cell Signaling Technology (2595S)), XPD (D3Z6I) (Cell Signaling Technology (11963)), NTH1 (Abcam (ab236912)), MUTYH (Abcam (ab228722)), FANCJ (BACH1/BRIP1) (Cell Signaling Technology (4578)), POLD1 (BD Biosciences (610972)), FTH^46^, TFR1 (Thermo Fisher (13–6800)), HSP60 (Cell Signaling Technology (12165S)), AFG3L2 (Proteintech (14631-1-AP)), HSP90 (Cell Signaling Technology (4877), Tubulin (Cell Signaling Technology (12351)), β-Actin (Sigma Aldrich (A3854) C/EBPβ Antibody (Cell Signaling Technology #9008),Id2 (D39E8) (Cell Signaling Technology #3431) and RPA32/RPA2 Antibody (Cell Signaling Technology #52448).

### Real-time RT-PCR

RT-PCR was performed as previously described^2^. Primers used were: human SFXN4 forward: GTCCAGATGAAGTATGGCCTGA; human SFXN4 reverse: CCCAGGACATTGCCTTCCTT; human β-Actin forward: TTG CCG ACA GGA TGC AGA AGG A; human β-Actin reverse: AGG TGG ACA GCG AGG CCA GGA T.

### Seahorse extracellular flux analysis

Oxygen consumption rate and extracellular acidification rate were measured using a Seahorse XF 96 Analyzer (Agilent) with an Agilent Seahorse XF Cell Mito Stress Test Kit^16^.

### Fe-S cluster fluorescent assay

Fe-S cluster fluorescent assay was performed as described^47^. Fe-S fluorescent sensor plasmids were a generous gift of Dr. Jonathan J Silberg, Department of Biochemistry and Cell biology, Rice University, USA. N- and C-terminal Venus-GRX2 fusion constructs were mixed in 1:1 v/v ratio and co-transfected into SFXN4 KO or control cells using FugeneHD (Promega). After 24 h, fluorescence was quantified by flow cytometry.

### Cell viability

3 × 10^3^ cells were seeded in 96 well plates and treated with the following reagents: cisplatin (Sigma Alderich), Rucaparib and Olaparib (Selleckchem). Cell viability was assessed 24–72 h post-treatment using calcein-AM (Millipore).

### Live/dead cell assay

Cell viability and death was assessed according to the manufacturer’s protocol using a live/dead cell assay (ThermoFisher Scientific).

### Flow cytometry

Single cell suspensions labeled with fluorescent indicators were analyzed on a BD LSRII flow cytometer (BD Biosciences). FCS list mode data was analyzed using FlowJo software (BD Biosciences).

### Mitochondrial superoxide

Cells were loaded with 5 µM MitoSOX Red mitochondrial superoxide indicator (Thermo Fisher Scientific) for 45 min at 37 °C in a humidified incubator at 5% CO_2_. Then cells were washed with PBS (GIBCO) and analyzed by flow cytometry or fixed with 4% paraformaldehyde (Thermo Fisher Scientific) and mounted using ProLong Gold anti-fade reagent (Invitrogen). Images were acquired using inverted microscopy (Zeiss Axio Vert.A1).

### Cellular reactive oxygen species

Cells were treated with 5 µM CellRox Deep Red Reagent (Thermoscietific) for 30 min at 37 °C in a humidified incubator at 5% CO_2_. Cells were then washed with PBS (GIBCO) and analyzed by flow cytometry.

### Detection of ferrous iron in mitochondria

Cells were treated with Mito-FerroGreen (ThermoFisher (Dojindo Molecular Technologies Inc)) for 2 h at 37 °C in a humidified incubator at 5% CO_2_. Then cells were washed with PBS (GIBCO) and analyzed by flow cytometry.

### Immunofluorescent staining of foci and cisplatin adducts and flow cytometry analysis

Cells were treated with cisplatin (Sigma Aldrich) or left untreated for 24–48 h, fixed with paraformaldehyde, permeabilized using 0.1% Triton X-100 (Sigma), and then blocked with 5% BSA at room temperature for 2 h. Anti-human P-Histone H2A.X (Cell Signaling Technology (9718S), anti-human RAD51 (Invitrogen (MA1-23271)), anti-human Cisplatin Modified DNA Antibody (CP9/19) (Novus biologicals (NBP2-50165)) and Alexa Fluor 555 conjugated secondary antibodies were applied at 1:400 to 1:800 dilutions for one hour. After extensive washes with PBS, cells were analyzed using flow cytometry or slides were mounted with ProLong Gold anti-fade reagent (Invitrogen). Images were acquired using inverted microscopy (Zeiss Axio Vert.A1).

### Host cell reactivation assay

A reporter plasmid containing Firefly luciferase (pGL3 (Promega)) was treated ex vivo with 0, 20, 100, or 500 µM cisplatin for 24 h at 37 °C and DNA damage assessed using a host cell reactivation assay as described^37^.

### Animal experiments

All animal studies were conducted in accordance with the recommendations in the Guide for the Care and Use of Laboratory Animals of the Association for the Assessment and Accreditation of Laboratory Animal Care International (AAALAC). The experimental protocol was approved prior to initiation of the study by the Institutional Animal Care and Use Committee (IACUC) at the University of Connecticut Health Center (protocol # 101941). Female NOD.Cg-Prkdcscid Il2rgtm1Wjl/SzJ mice (NSG; ~ 6 weeks of age) were obtained from Jackson Laboratory. Mice were injected intraperitoneally (i.p) with either 2 × 10^5^ FT^T^ control cells (CRISPR scrambled cells), 2 × 10^5^ FT^T^ SFXN4 KO cells, or 2 × 10^6^ FT^T^ SFXN4 KO cells. A 1:1 ratio of cells in PBS to matrigel (Corning Ref# 354230) was used, with each mouse receiving a total of 200 µl of cell/matrigel suspension. Four mice were used in each group, with group size selected based on previous experience with this tumor model^2^. Tumor number was the primary outcome measure. Mice were randomly allocated to treatment groups; however, no explicit protocol for randomization was used. No a priori criteria were established for excluding animals from the study; however, one mouse injected with 2 × 10^6^ FT^T^ SFXN4 KO cells was excluded from the analysis due to accidental injection of tumor cells into an inappropriate site (muscle tissue; confirmed by P.H., a board-certified pathologist). 23 days post injection, animals were sacrificed, tumors were counted and the combined weight of all tumors within the abdomen of each mouse was measured. Differences between tumor number and weight in mice bearing control or SFXN4 knockout tumors were assessed using Students two-tailed t test, with p ≤ 0.05 accepted as significant. Data were normally distributed. Results of this study are reported according to ARRIVE guidelines^48^.

### RAD51 quantification in fixed tumors

Tumors isolated from NSG mice were sectioned and verified by P.H., a board-certified pathologist. Sections of formalin-fixed, paraffin-embedded tissues were immunostained with antibody to human RAD51 (ThermoFisher scientific cat# PA5-27195) followed by anti-Rabbit secondary antibody (Biocare Medical MACH2 Rabbit HRP-Polymer; catalog # RHRP520#) and staining with 3,3'-diaminobenzidine (Biocare Medical). Gill”s Hematoxylin III and Lithium Blue (Poly Scientific) were used as counterstains. Nuclear staining was quantified using ImageJ. Percent RAD51 positive nuclei were quantified in 3 random fields per tumor (each field containing approximately 150–200 cells), and the average percent (mean ± std deviation) RAD51 positive nuclei calculated for each tumor.

### Statistics

Experiments were performed at least in triplicate (biological replicates) with 3–8 technical replicates per point. Significance was assessed using Students two-tailed t test, with p ≤ 0.05 accepted as significant. To compare dose responses between drug type and cell types, log-logistic models were implemented using the drc package^49^ (v 3.0-1^49^) in R v 4.1.0^50^. Two-parameter models were used where the minimum and maximum values of the curve were set to 0 and 1 respectively. Differences in IC50 values from these curves were estimated using the compParm function. Each combination of cell type and drug were replicated 3 times, resulting in 3 estimates of IC50 per cell and drug combination. Pooled estimates of overall differences in IC50 across experiments were combined using inverse variance weighting and significance assessed using z-tests, with the null hypothesis that the pooled difference = 0.

## Supplementary Information

Supplementary Information.

## Data Availability

The data generated in this study are available upon request from the corresponding author.
