# Supplementary material for: Complementary anti-cancer pathways triggered by inhibition of sideroflexin 4 in ovarian cancer
Source: Sci Rep. 2022 Nov 19;12:19936. doi: 10.1038/s41598-022-24391-3 (PMC9675821; doi:10.1038/s41598-022-24391-3)
Supplement: Supplementary file 1 — Supplementary Information. [file 41598_2022_24391_MOESM1_ESM.pptx]

## Slide 1
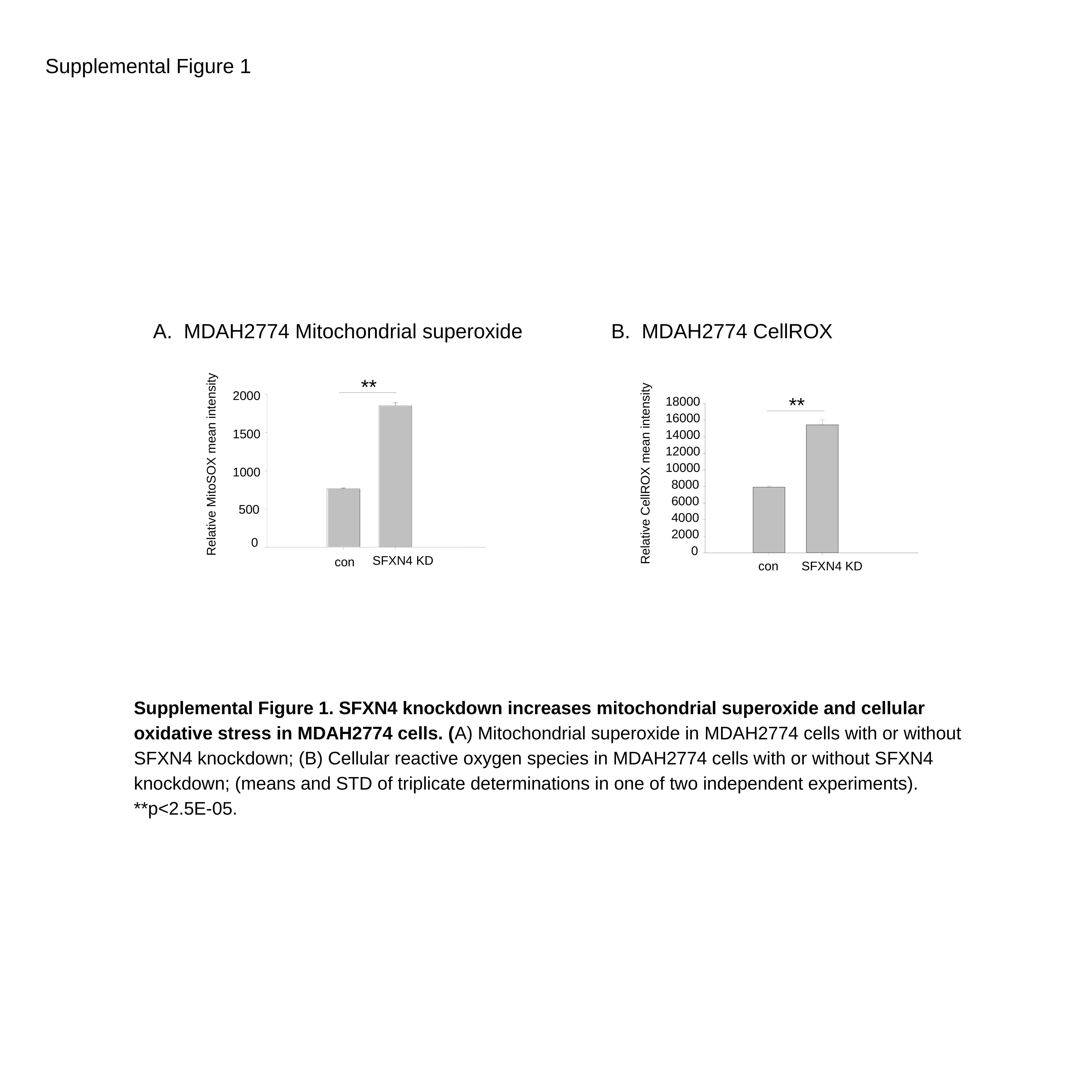

Supplemental Figure 1
A. MDAH2774 Mitochondrial superoxide
B. MDAH2774 CellROX
**
2000
**
18000
16000
1500
14000
12000
Relative MitoSOX mean intensity
10000
1000
Relative CellROX mean intensity
8000
6000
500
4000
2000
0
0
SFXN4 KD
con
SFXN4 KD
con
Supplemental Figure 1. SFXN4 knockdown increases mitochondrial superoxide and cellular oxidative stress in MDAH2774 cells. (A) Mitochondrial superoxide in MDah2774 cells with or without SFXN4 knockdown; (B) Cellular reactive oxygen species in MDAH2774 cells with or without SFXN4 knockdown; (means and STD of triplicate determinations in one of two independent experiments). **p<2.5E-05.

## Slide 2
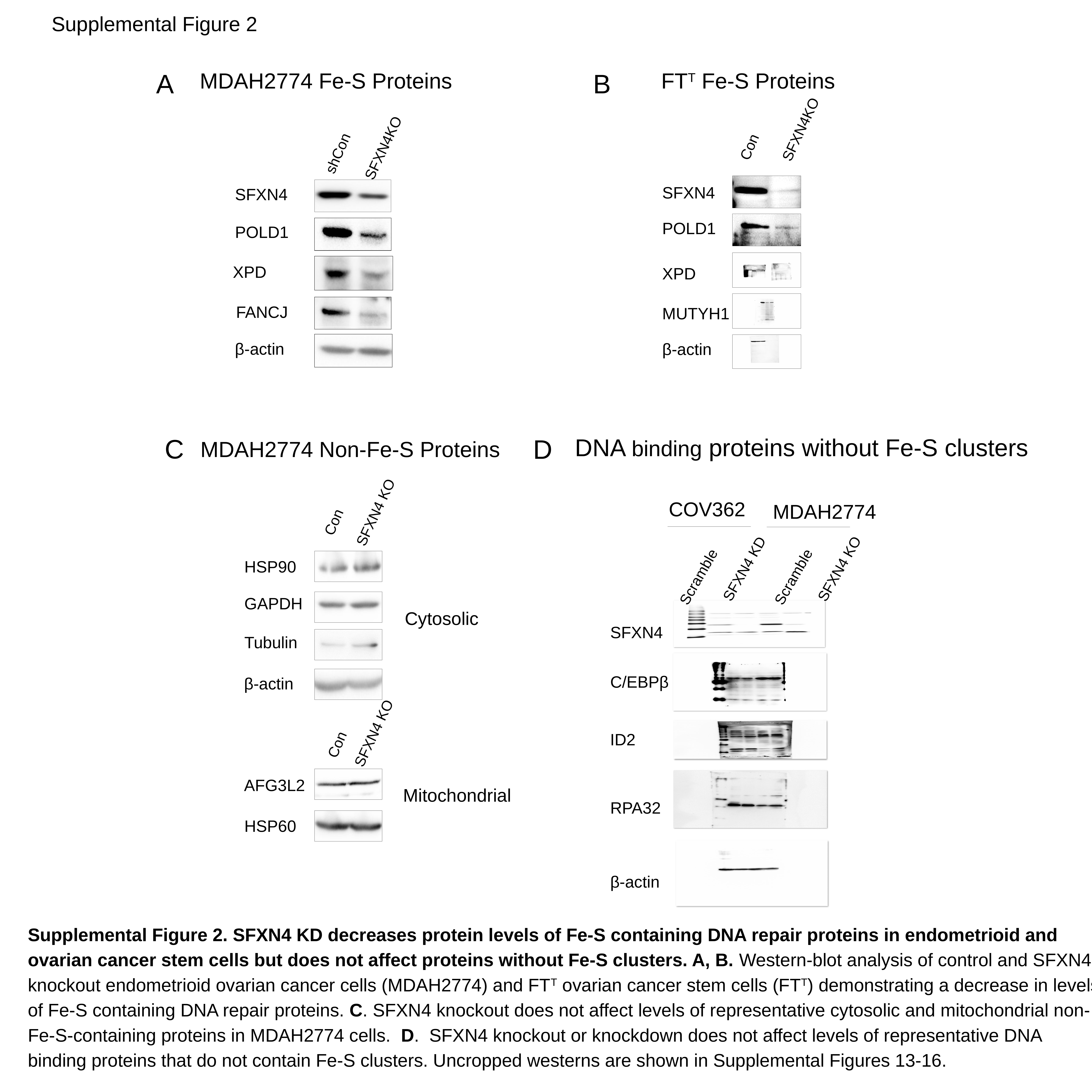

Supplemental Figure 2
A
MDAH2774 Fe-S Proteins
B
FTT Fe-S Proteins
SFXN4KO
Con
SFXN4KO
shCon
SFXN4
SFXN4
POLD1
POLD1
XPD
XPD
FANCJ
MUTYH1
β-actin
β-actin
C
DNA binding proteins without Fe-S clusters
D
MDAH2774 Non-Fe-S Proteins
COV362
MDAH2774
SFXN4 KO
Con
HSP90
SFXN4 KD
SFXN4 KO
Scramble
Scramble
GAPDH
Cytosolic
SFXN4
Tubulin
C/EBPβ
β-actin
SFXN4 KO
ID2
Con
AFG3L2
Mitochondrial
RPA32
HSP60
β-actin
Supplemental Figure 2. SFXN4 KD decreases protein levels of Fe-S containing DNA repair proteins in endometrioid and ovarian cancer stem cells but does not affect proteins without Fe-S clusters. A, B. Western-blot analysis of control and SFXN4 knockout endometrioid ovarian cancer cells (MDAH2774) and FTT ovarian cancer stem cells (FTT) demonstrating a decrease in levels of Fe-S containing DNA repair proteins. C. SFXN4 knockout does not affect levels of representative cytosolic and mitochondrial non-Fe-S-containing proteins in MDAH2774 cells. D. SFXN4 knockout or knockdown does not affect levels of representative DNA binding proteins that do not contain Fe-S clusters. Uncropped westerns are shown in Supplemental Figures 13-16.

## Slide 3
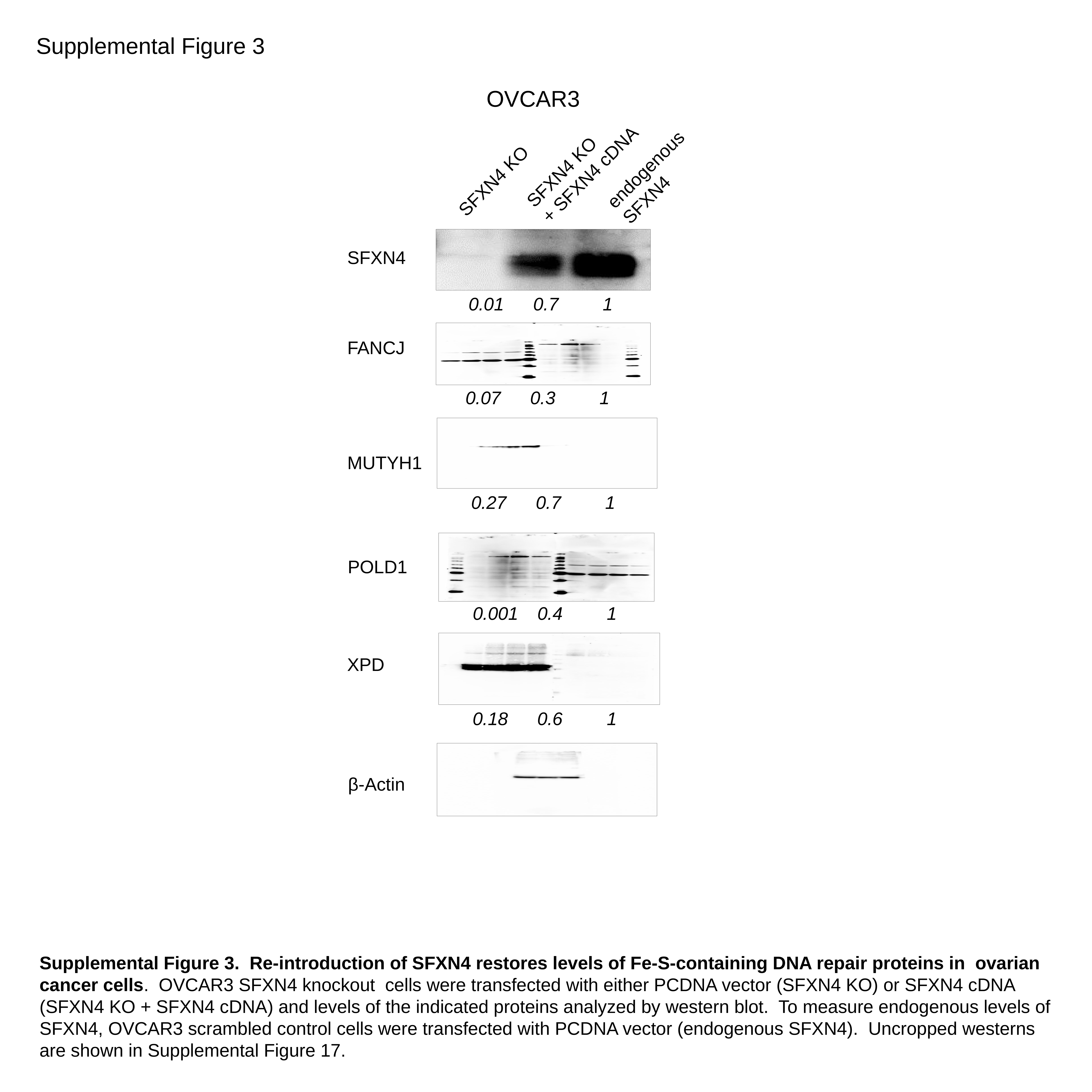

Supplemental Figure 3
OVCAR3
endogenous SFXN4
SFXN4 KO
+ SFXN4 cDNA
SFXN4 KO
SFXN4
0.01
0.7
1
FANCJ
0.07
0.3
1
MUTYH1
0.27
0.7
1
POLD1
0.001
0.4
1
XPD
0.18
0.6
1
β-Actin
Supplemental Figure 3. Re-introduction of SFXN4 restores levels of Fe-S-containing DNA repair proteins in ovarian cancer cells. OVCAR3 SFXN4 knockout cells were transfected with either PCDNA vector (SFXN4 KO) or SFXN4 cDNA (SFXN4 KO + SFXN4 cDNA) and levels of the indicated proteins analyzed by western blot. To measure endogenous levels of SFXN4, OVCAR3 scrambled control cells were transfected with PCDNA vector (endogenous SFXN4). Uncropped westerns are shown in Supplemental Figure 17.

## Slide 4
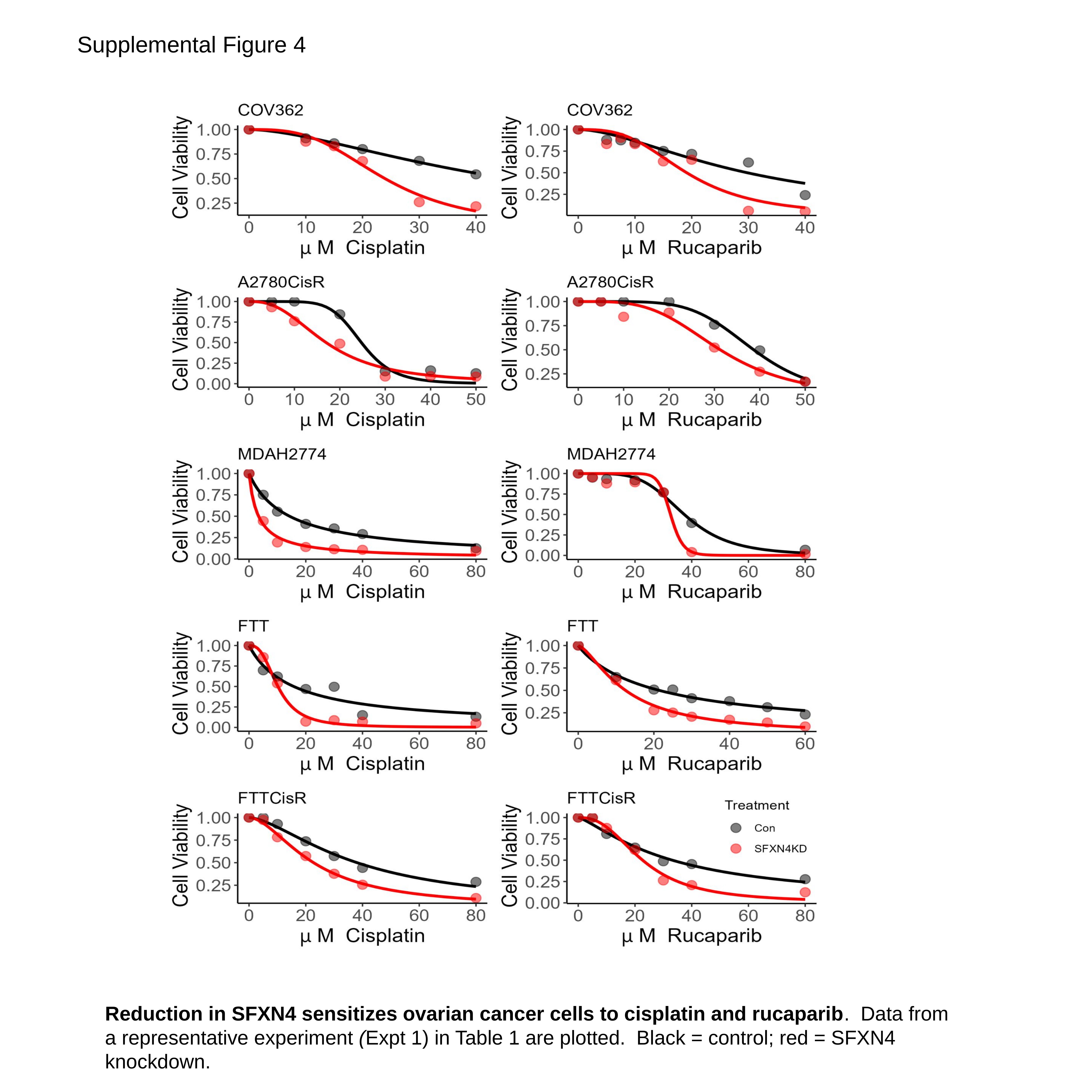

# Supplemental Figure 4
Reduction in SFXN4 sensitizes ovarian cancer cells to cisplatin and rucaparib. Data from a representative experiment (Expt 1) in Table 1 are plotted. Black = control; red = SFXN4 knockdown.

## Slide 5
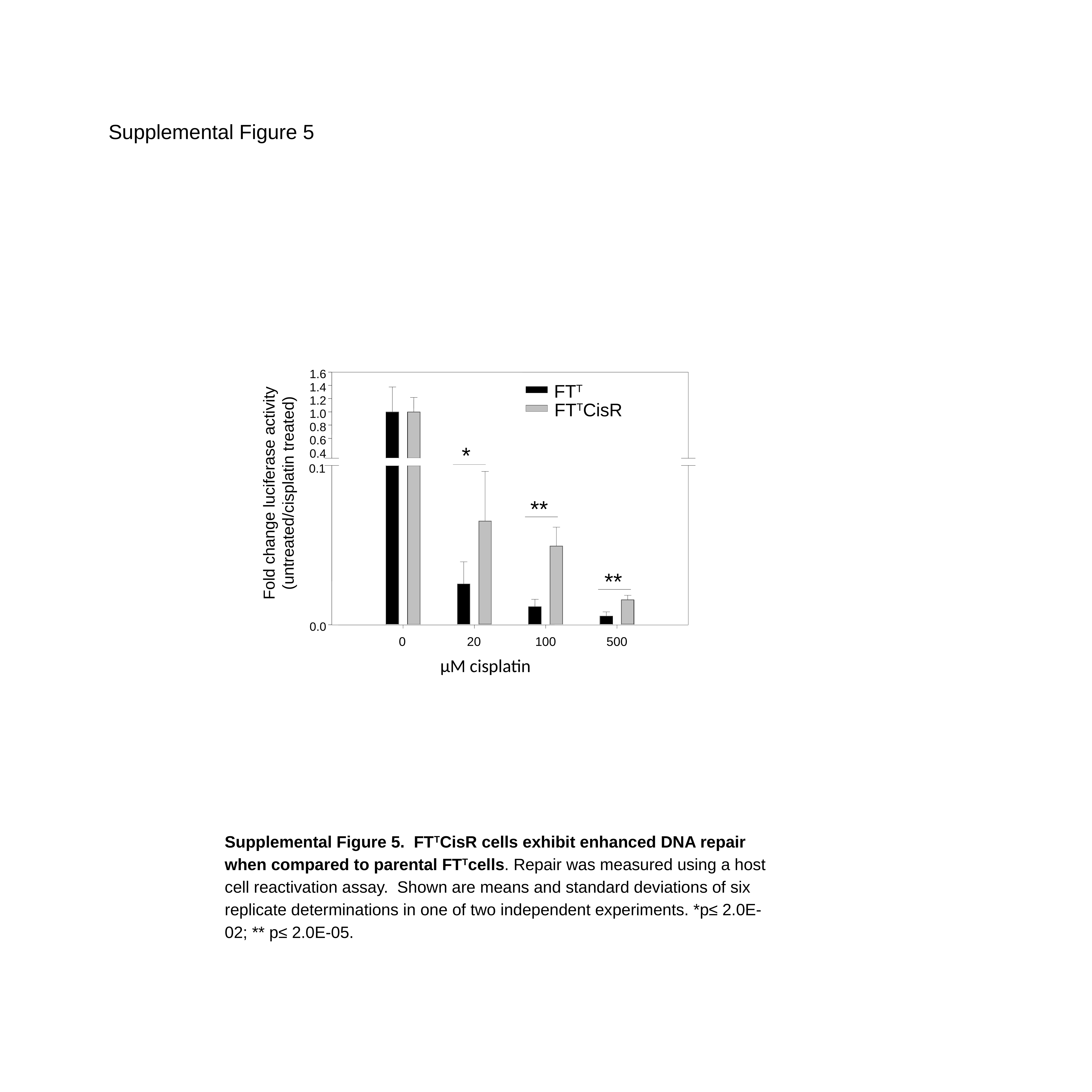

Supplemental Figure 5
1.6
FTT
1.4
1.2
FTTCisR
1.0
0.8
0.6
*
0.4
0.1
Fold change luciferase activity
(untreated/cisplatin treated)
**
**
0.0
0
20
100
500
µM cisplatin
Supplemental Figure 5. FTTCisR cells exhibit enhanced DNA repair when compared to parental FTTcells. Repair was measured using a host cell reactivation assay. Shown are means and standard deviations of six replicate determinations in one of two independent experiments. *p≤ 2.0E-02; ** p≤ 2.0E-05.

## Slide 6
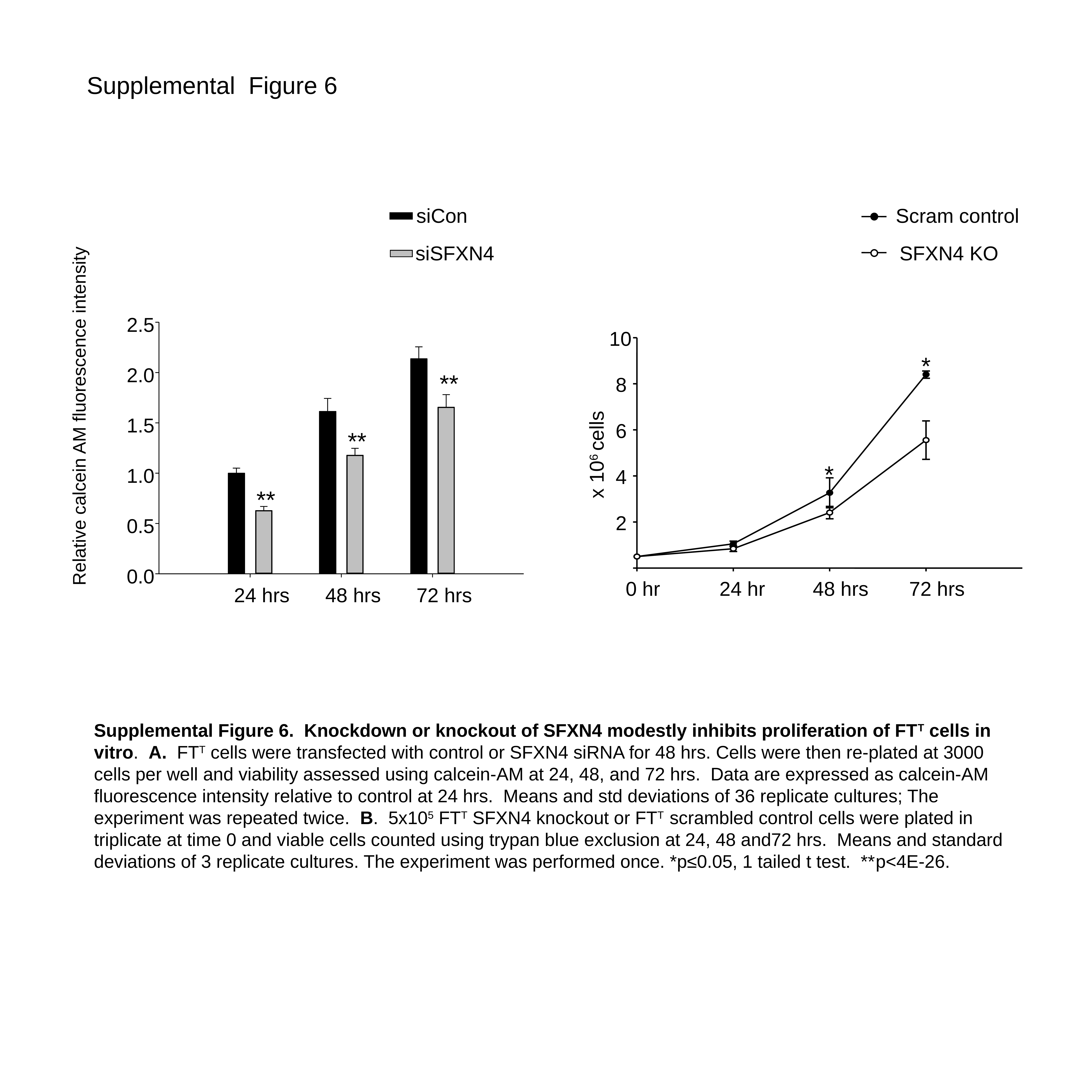

Supplemental Figure 6
siCon
 Scram control
siSFXN4
SFXN4 KO
2.5
10
8
6
 x 106 cells
4
2
0 hr
24 hr
48 hrs
72 hrs
2.0
**
Relative calcein AM fluorescence intensity
1.5
**
1.0
**
0.5
0.0
24 hrs
48 hrs
72 hrs
*
*
Supplemental Figure 6. Knockdown or knockout of SFXN4 modestly inhibits proliferation of FTT cells in vitro. A. FTT cells were transfected with control or SFXN4 siRNA for 48 hrs. Cells were then re-plated at 3000 cells per well and viability assessed using calcein-AM at 24, 48, and 72 hrs. Data are expressed as calcein-AM fluorescence intensity relative to control at 24 hrs. Means and std deviations of 36 replicate cultures; The experiment was repeated twice. B. 5x10^5^ FTT SFXN4 knockout or FTT scrambled control cells were plated in triplicate at time 0 and viable cells counted using trypan blue exclusion at 24, 48 and72 hrs. Means and standard deviations of 3 replicate cultures. The experiment was performed once. *p≤0.05, 1 tailed t test. **p<4E-26.

## Slide 7
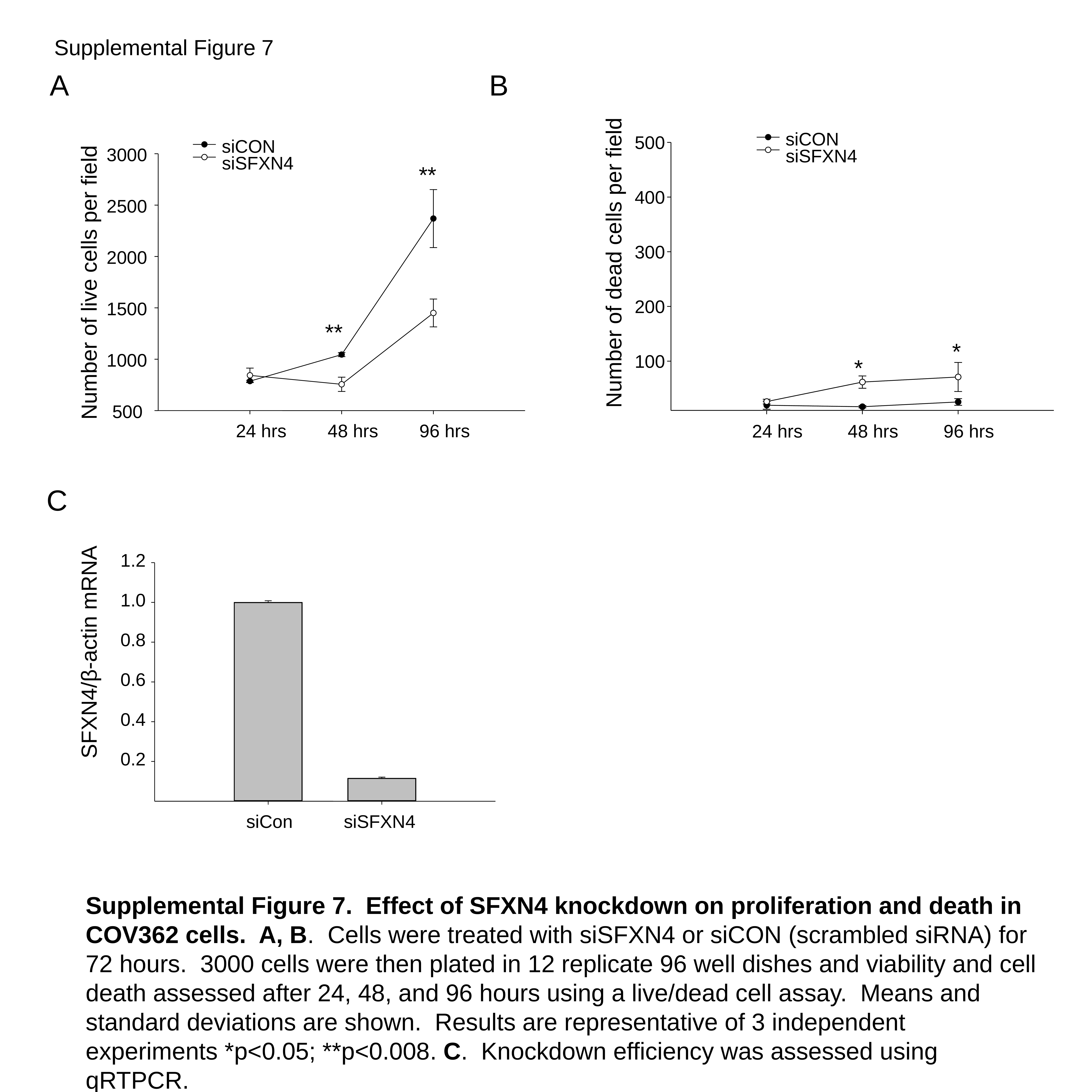

Supplemental Figure 7
A
B
siCON
siSFXN4
500
siCON
siSFXN4
3000
**
400
2500
Number of dead cells per field
300
2000
Number of live cells per field
200
1500
**
*
1000
100
*
500
24 hrs
48 hrs
96 hrs
24 hrs
48 hrs
96 hrs
C
1.2
1.0
 SFXN4/β-actin mRNA
0.8
0.6
0.4
0.2
siCon
siSFXN4
Supplemental Figure 7. Effect of SFXN4 knockdown on proliferation and death in COV362 cells. A, B. Cells were treated with siSFXN4 or siCON (scrambled siRNA) for 72 hours. 3000 cells were then plated in 12 replicate 96 well dishes and viability and cell death assessed after 24, 48, and 96 hours using a live/dead cell assay. Means and standard deviations are shown. Results are representative of 3 independent experiments *p<0.05; **p<0.008. C. Knockdown efficiency was assessed using qRTPCR.

## Slide 8
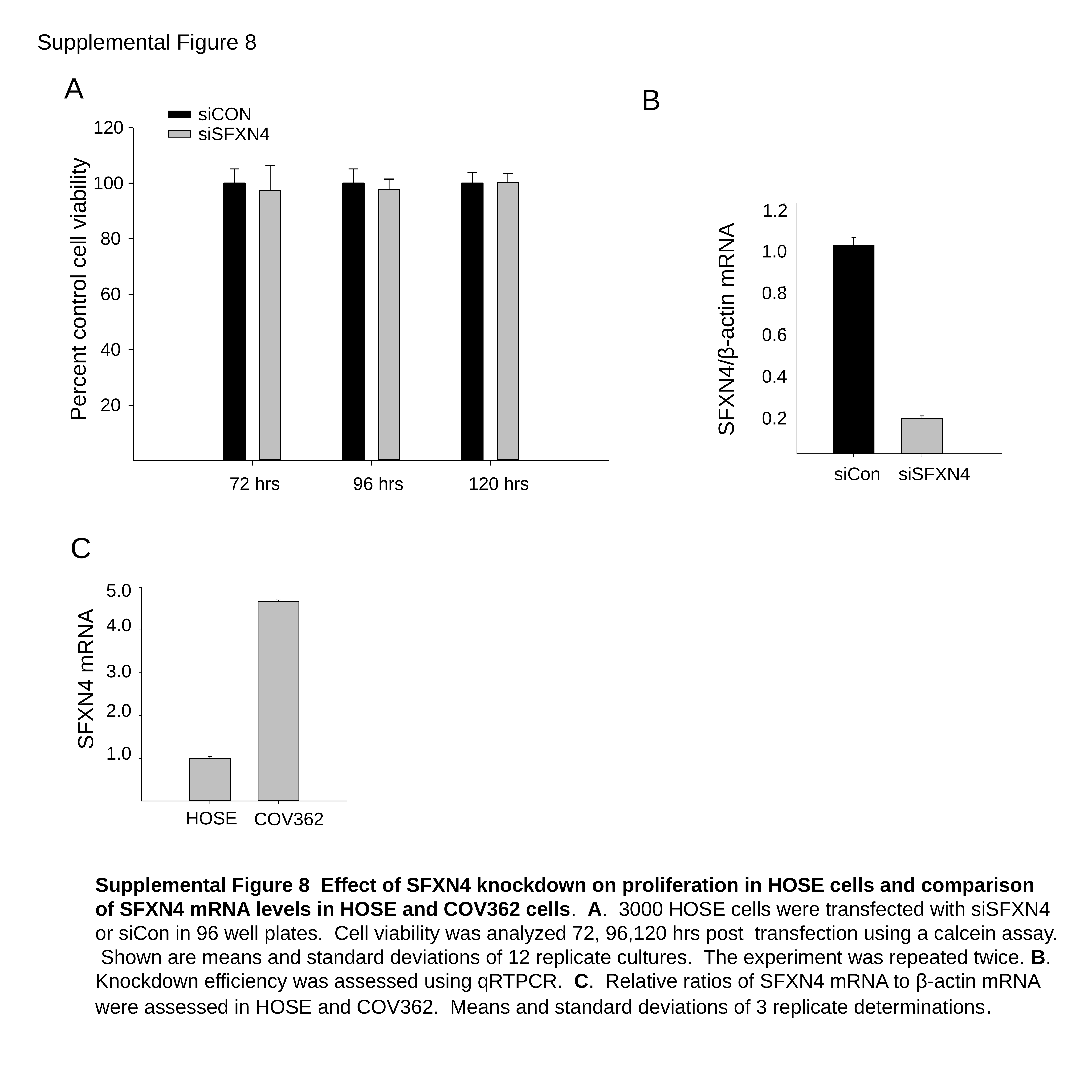

Supplemental Figure 8
A
B
siCON
120
siSFXN4
100
1.2
80
1.0
Percent control cell viability
0.8
60
 SFXN4/β-actin mRNA
0.6
40
0.4
20
0.2
siCon
siSFXN4
72 hrs
96 hrs
120 hrs
C
5.0
4.0
3.0
 SFXN4 mRNA
2.0
1.0
HOSE
COV362
Supplemental Figure 8 Effect of SFXN4 knockdown on proliferation in HOSE cells and comparison of SFXN4 mRNA levels in HOSE and COV362 cells. A. 3000 HOSE cells were transfected with siSFXN4 or siCon in 96 well plates. Cell viability was analyzed 72, 96,120 hrs post transfection using a calcein assay. Shown are means and standard deviations of 12 replicate cultures. The experiment was repeated twice. B. Knockdown efficiency was assessed using qRTPCR. C. Relative ratios of SFXN4 mRNA to β-actin mRNA were assessed in HOSE and COV362. Means and standard deviations of 3 replicate determinations.

## Slide 9
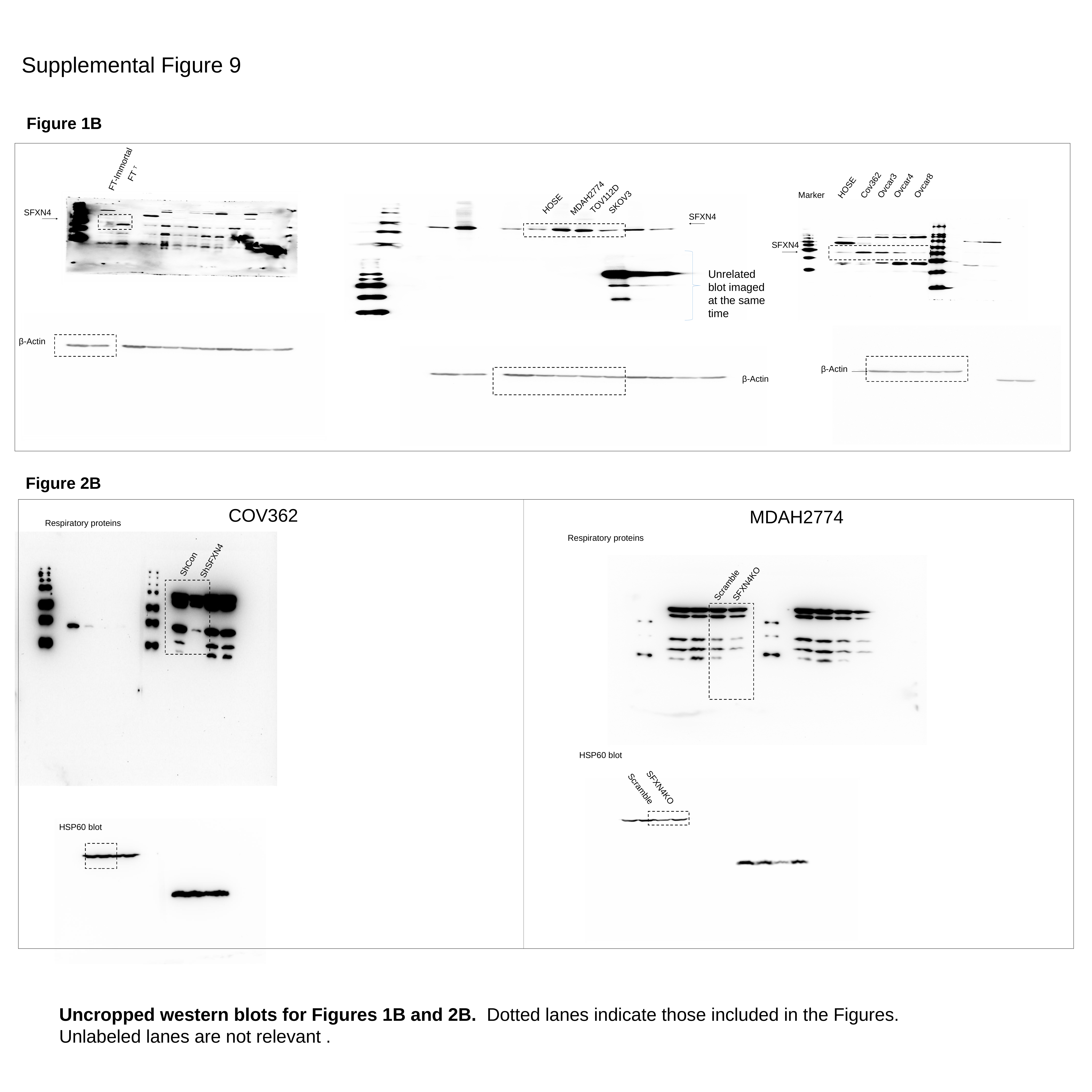

Supplemental Figure 9
Figure 1B
FT-Immortal
FT T
Cov362
Ovcar8
Ovcar3
Ovcar4
HOSE
Marker
MDAH2774
KD
TOV112D
SKOV3
HOSE
50
SFXN4
SFXN4
40
30
20
SFXN4
Unrelated blot imaged at the same time
β-Actin
β-Actin
β-Actin
Figure 2B
COV362
MDAH2774
Respiratory proteins
Respiratory proteins
ShSFXN4
ShCon
SFXN4KO
Scramble
HSP60 blot
SFXN4KO
Scramble
HSP60 blot
Uncropped western blots for Figures 1B and 2B. Dotted lanes indicate those included in the Figures. Unlabeled lanes are not relevant .

## Slide 10
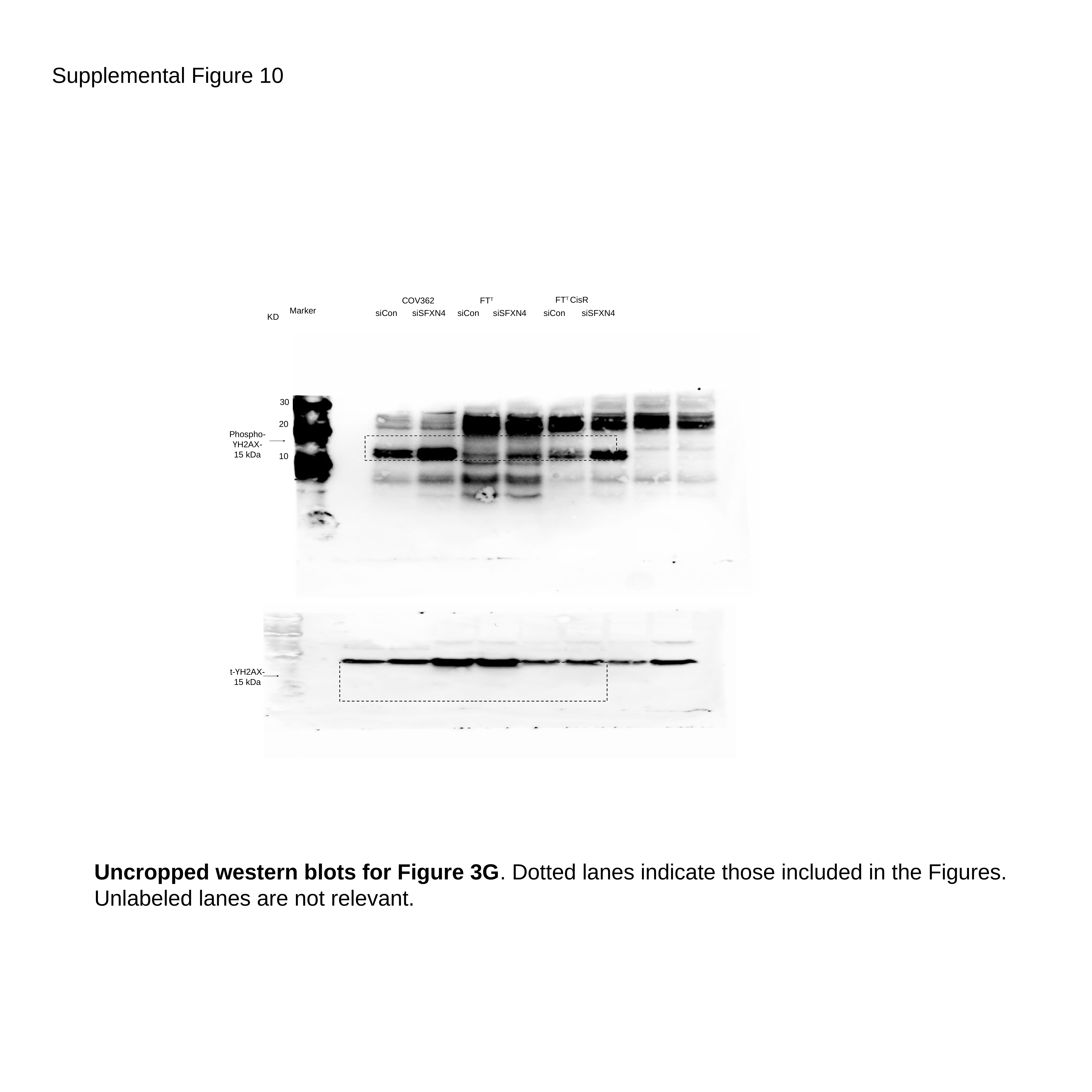

Supplemental Figure 10
FTT CisR
COV362
FTT
Marker
siCon
siSFXN4
siCon
siSFXN4
siCon
siSFXN4
KD
30
20
Phospho-ҮH2AX-
15 kDa
10
t-ҮH2AX-
15 kDa
Uncropped western blots for Figure 3G. Dotted lanes indicate those included in the Figures. Unlabeled lanes are not relevant.

## Slide 11
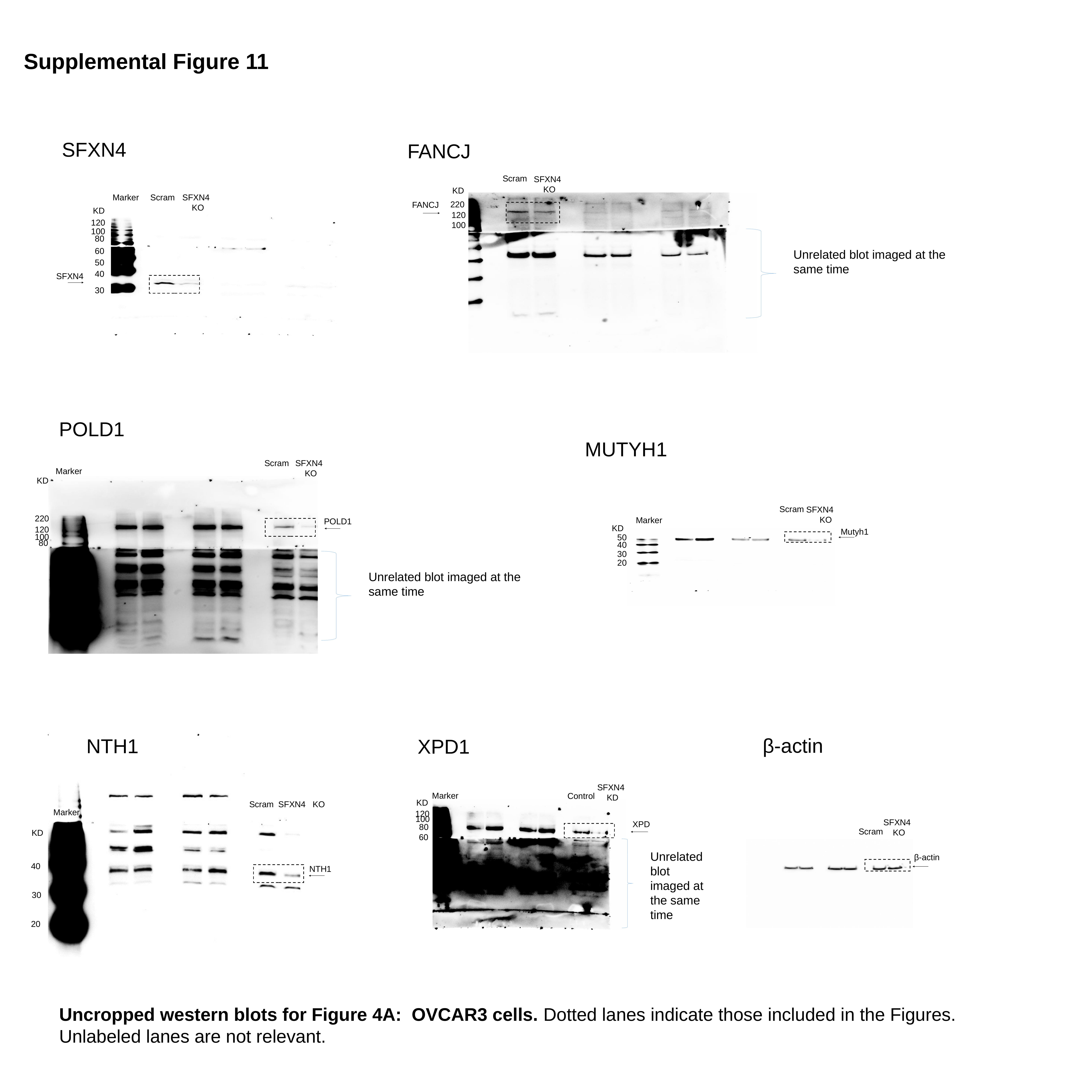

Supplemental Figure 11
SFXN4
FANCJ
Scram
SFXN4
 KO
KD
Marker
Scram
SFXN4
 KO
220
FANCJ
KD
120
120
100
100
80
60
Unrelated blot imaged at the same time
50
40
SFXN4
30
POLD1
MUTYH1
Scram
SFXN4
 KO
Marker
KD
220
POLD1
120
100
80
Scram
SFXN4 KO
Marker
KD
Mutyh1
50
40
30
20
Unrelated blot imaged at the same time
Scram
SFXN4 KO
Marker
KD
40
NTH1
30
20
β-actin
NTH1
XPD1
SFXN4
 KD
Marker
Control
KD
120
100
XPD
80
60
SFXN4
 KO
Scram
β-actin
Unrelated blot imaged at the same time
Uncropped western blots for Figure 4A: OVCAR3 cells. Dotted lanes indicate those included in the Figures. Unlabeled lanes are not relevant.

## Slide 12
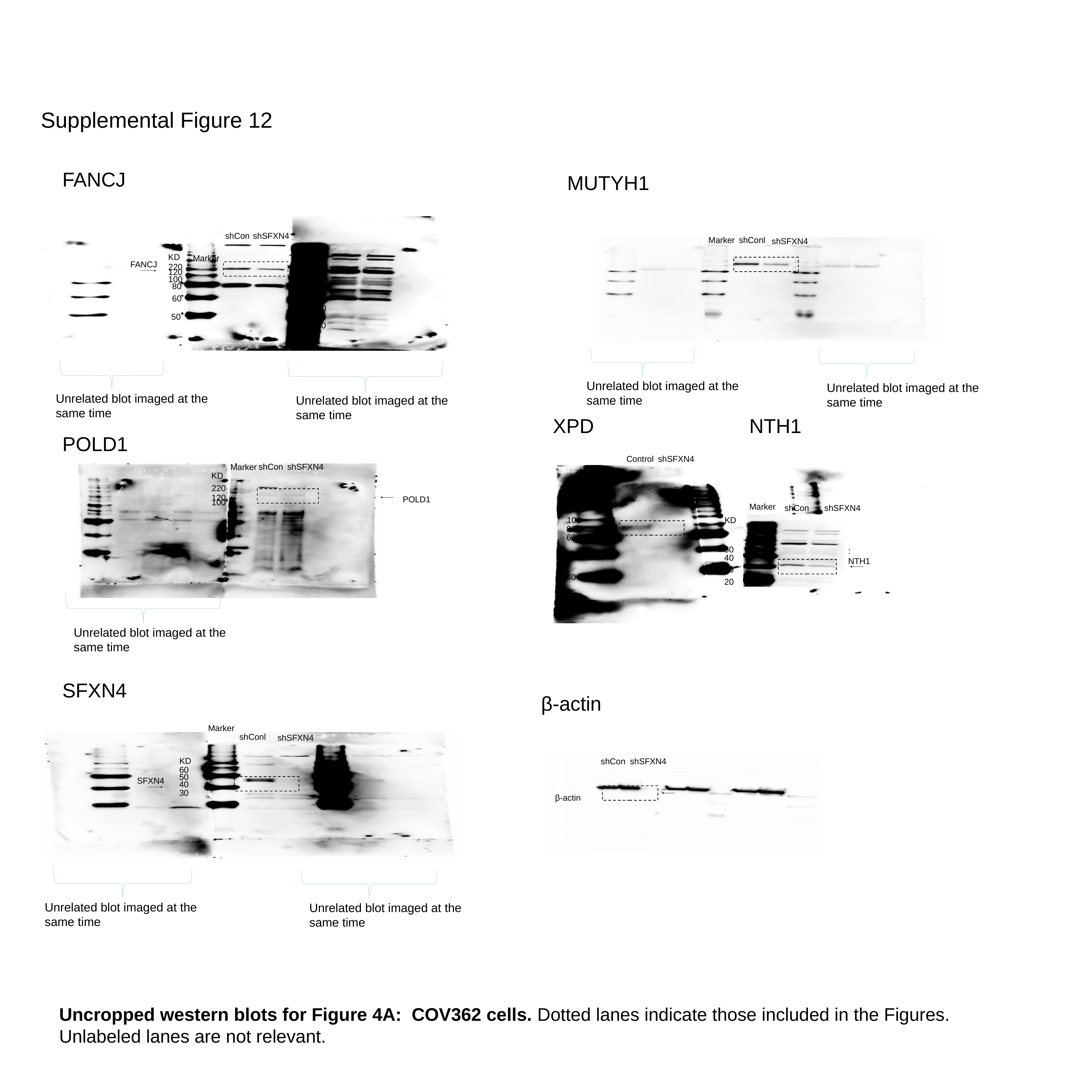

Supplemental Figure 12
FANCJ
MUTYH1
shCon
shSFXN4
KD
Marker
KD
FANCJ
220
120
50
100
40
80
30
60
20
50
10
Marker
shConl
shSFXN4
Unrelated blot imaged at the same time
Unrelated blot imaged at the same time
Unrelated blot imaged at the same time
Unrelated blot imaged at the same time
XPD
NTH1
POLD1
Control
shSFXN4
shCon
shSFXN4
Marker
KD
220
120
POLD1
100
Marker
KD
Marker
shCon
shSFXN4
100
KD
80
60
50
:
NTH1
50
40
30
40
20
30
Unrelated blot imaged at the same time
SFXN4
β-actin
Marker
shConl
shSFXN4
KD
shCon
shSFXN4
60
50
SFXN4
40
30
β-actin
Unrelated blot imaged at the same time
Unrelated blot imaged at the same time
Uncropped western blots for Figure 4A: COV362 cells. Dotted lanes indicate those included in the Figures. Unlabeled lanes are not relevant.

## Slide 13
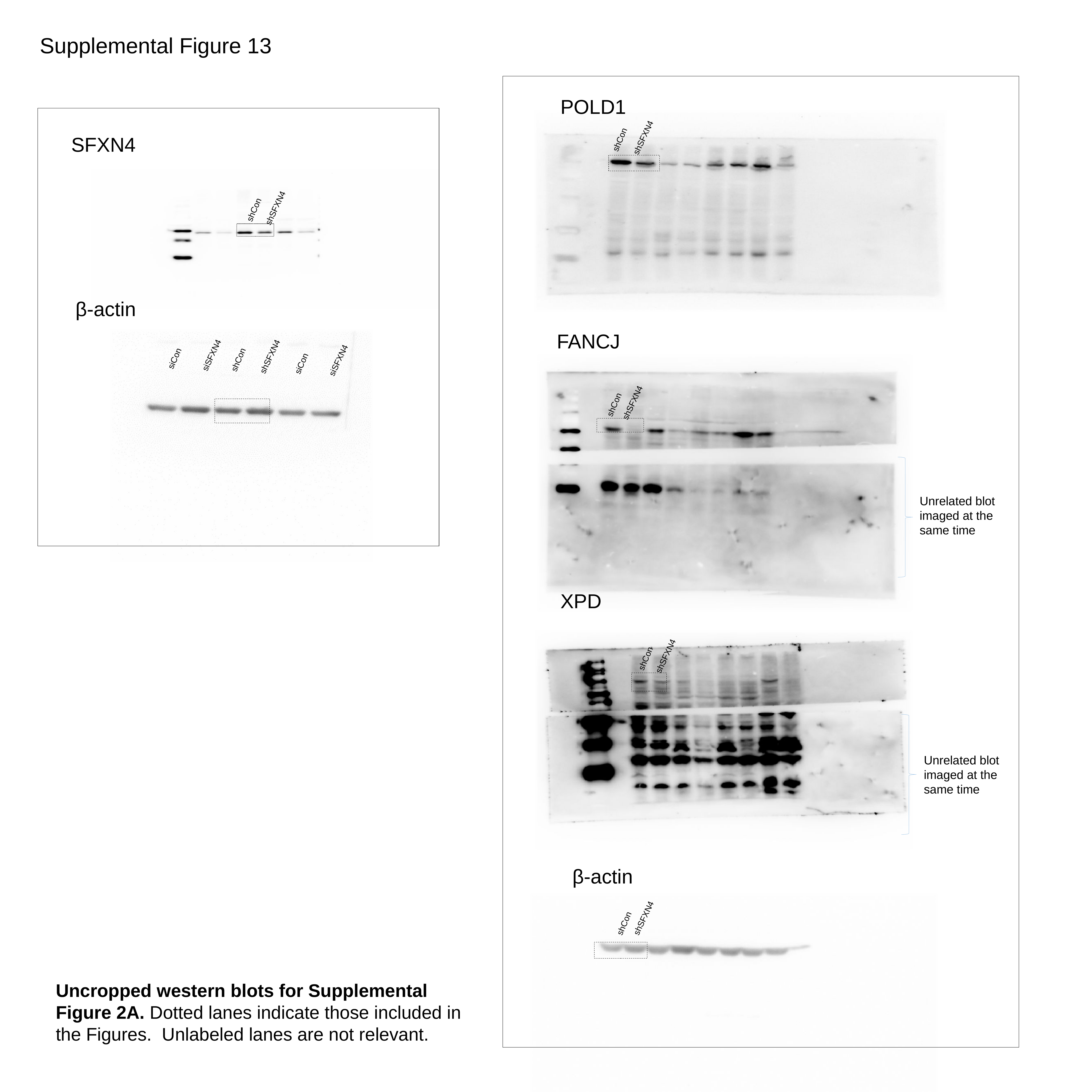

Supplemental Figure 13
POLD1
shSFXN4
shCon
SFXN4
shSFXN4
shCon
β-actin
FANCJ
siSFXN4
shSFXN4
siCon
shCon
siSFXN4
siCon
shSFXN4
shCon
Unrelated blot imaged at the same time
XPD
shSFXN4
shCon
Unrelated blot imaged at the same time
β-actin
shSFXN4
shCon
Uncropped western blots for Supplemental Figure 2A. Dotted lanes indicate those included in the Figures. Unlabeled lanes are not relevant.

## Slide 14
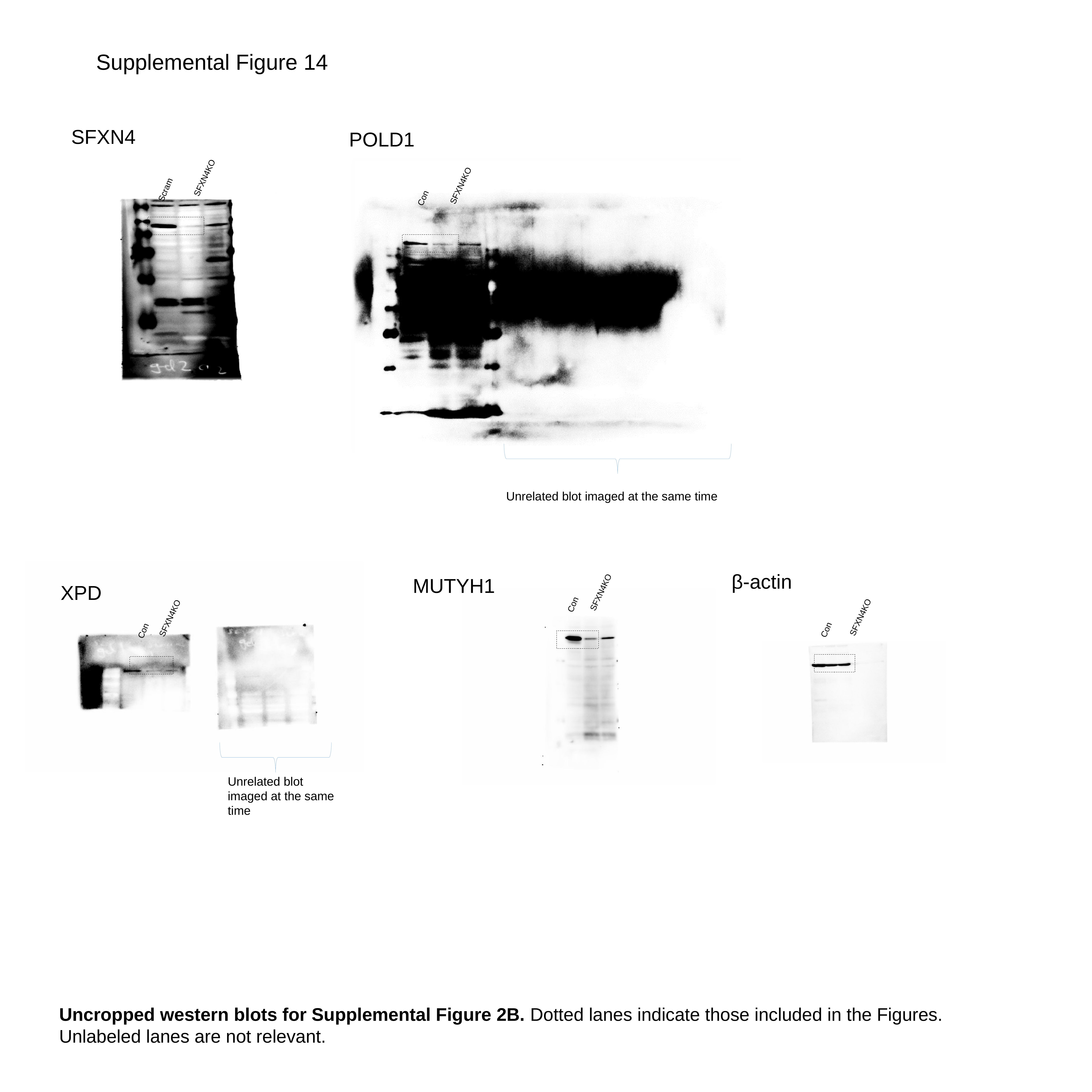

Supplemental Figure 14
SFXN4
POLD1
SFXN4KO
SFXN4KO
Scram
Con
Unrelated blot imaged at the same time
β-actin
MUTYH1
XPD
SFXN4KO
Con
SFXN4KO
SFXN4KO
Con
Con
Unrelated blot imaged at the same time
Uncropped western blots for Supplemental Figure 2B. Dotted lanes indicate those included in the Figures. Unlabeled lanes are not relevant.

## Slide 15
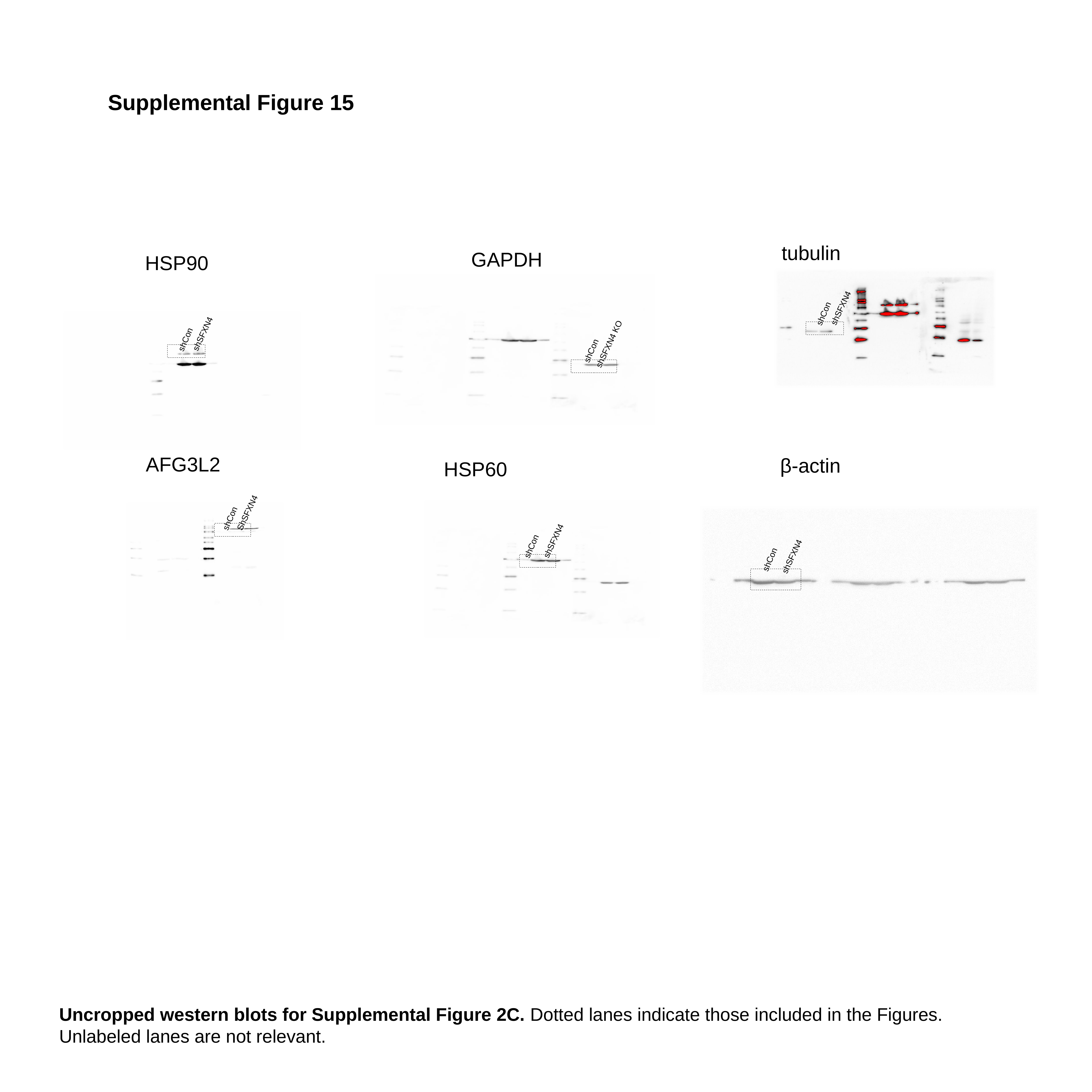

Supplemental Figure 15
tubulin
GAPDH
shSFXN4 KO
shCon
HSP90
shSFXN4
shCon
shSFXN4
shCon
AFG3L2
β-actin
HSP60
ShSFXN4
shCon
shSFXN4
shCon
shSFXN4
shCon
Uncropped western blots for Supplemental Figure 2C. Dotted lanes indicate those included in the Figures. Unlabeled lanes are not relevant.

## Slide 16
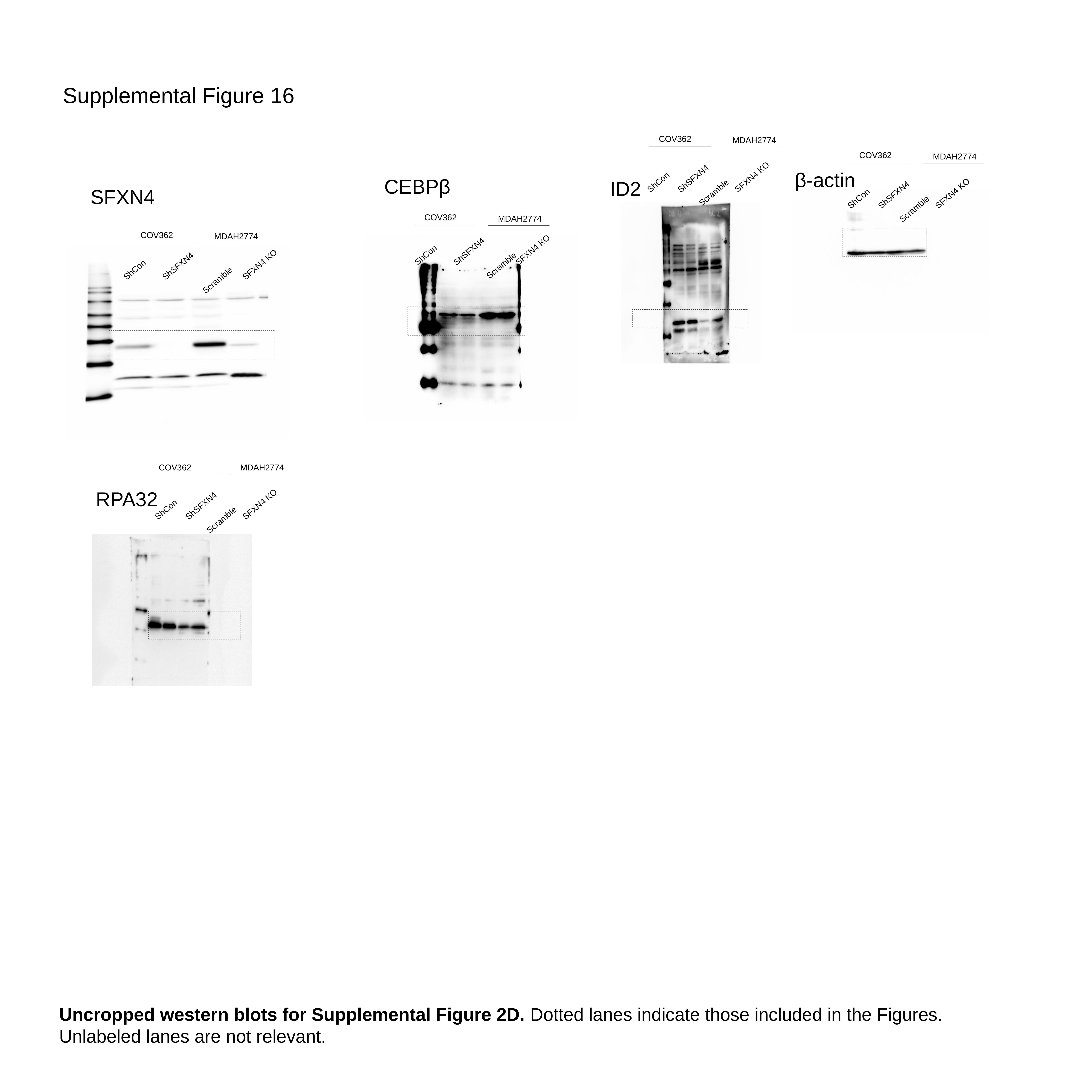

Supplemental Figure 16
COV362
MDAH2774
COV362
MDAH2774
β-actin
ShSFXN4
SFXN4 KO
CEBPβ
ID2
ShCon
SFXN4
ShSFXN4
SFXN4 KO
Scramble
ShCon
Scramble
COV362
MDAH2774
COV362
MDAH2774
ShSFXN4
SFXN4 KO
ShCon
ShSFXN4
SFXN4 KO
Scramble
ShCon
Scramble
Scramble
COV362
MDAH2774
RPA32
ShSFXN4
SFXN4 KO
ShCon
Scramble
Uncropped western blots for Supplemental Figure 2D. Dotted lanes indicate those included in the Figures. Unlabeled lanes are not relevant.

## Slide 17
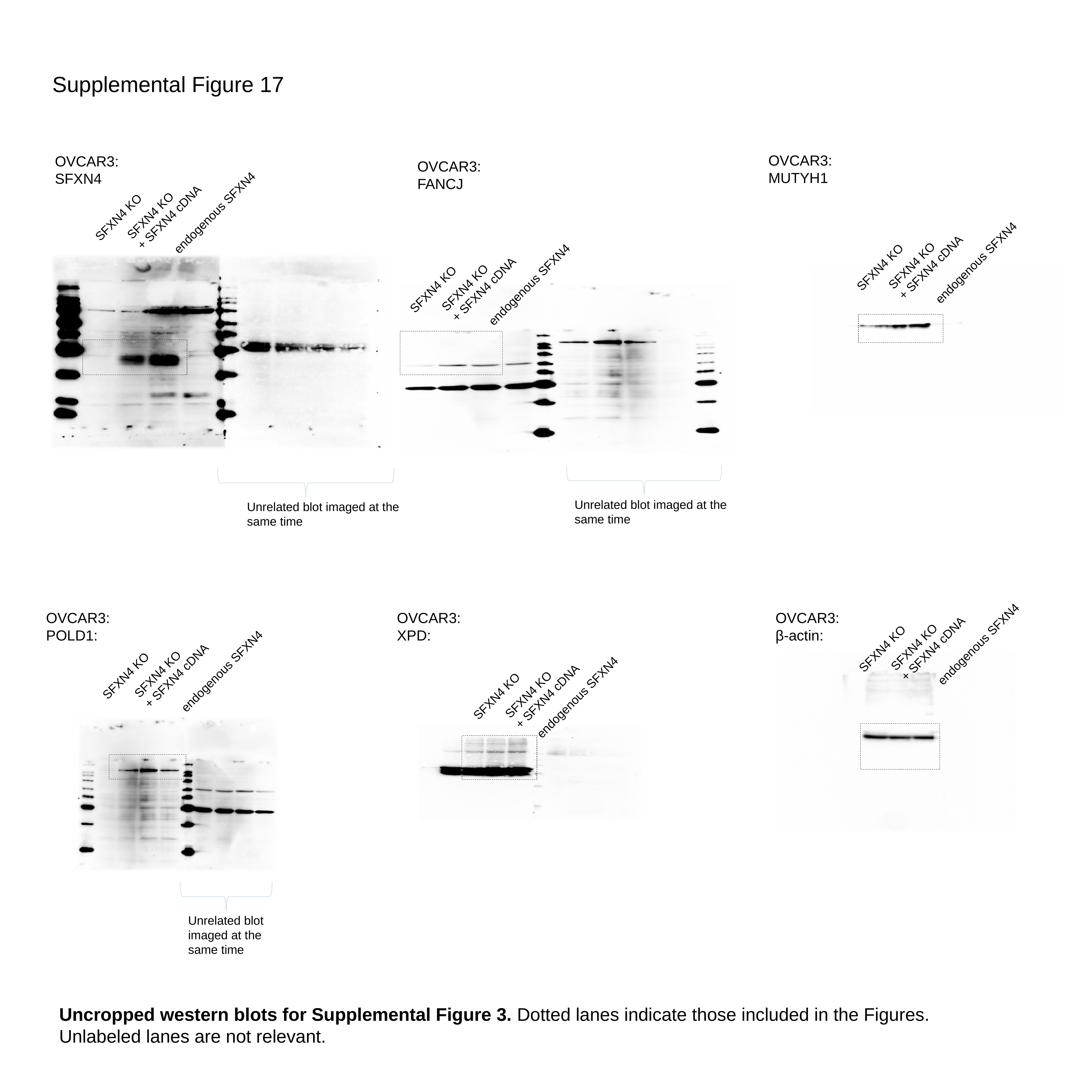

Supplemental Figure 17
OVCAR3:
MUTYH1
OVCAR3:
SFXN4
OVCAR3:
FANCJ
SFXN4 KO
SFXN4 KO
+ SFXN4 cDNA
endogenous SFXN4
SFXN4 KO
SFXN4 KO
+ SFXN4 cDNA
endogenous SFXN4
SFXN4 KO
SFXN4 KO
+ SFXN4 cDNA
endogenous SFXN4
Unrelated blot imaged at the same time
Unrelated blot imaged at the same time
OVCAR3:
POLD1:
OVCAR3:
XPD:
OVCAR3:
β-actin:
SFXN4 KO
SFXN4 KO
+ SFXN4 cDNA
endogenous SFXN4
SFXN4 KO
SFXN4 KO
+ SFXN4 cDNA
endogenous SFXN4
SFXN4 KO
SFXN4 KO
+ SFXN4 cDNA
endogenous SFXN4
Unrelated blot imaged at the same time
Uncropped western blots for Supplemental Figure 3. Dotted lanes indicate those included in the Figures. Unlabeled lanes are not relevant.
